# Supplementary material for: PIMT Controls Insulin Synthesis and Secretion through PDX1
Source: Int J Mol Sci. 2023 Apr 29;24(9):8084. doi: 10.3390/ijms24098084 (PMC10179560; doi:10.3390/ijms24098084)
Supplement: Supplementary file 1 [file ijms-24-08084-s001.zip › ijms-2177351-supplementary.pdf]

<u>**PIMT controls insulin synthesis and secretion through PDX1**</u>

Sharma R[1, #], Sujay K Maity[2], Partha Chakrabarti[2], Madhumohan R Katika[3], Satyamoorthy Kapettu[4], Kishore V. L. Parsa[1*] and Parimal Misra[1*]

<u>**Supplementary data**</u>

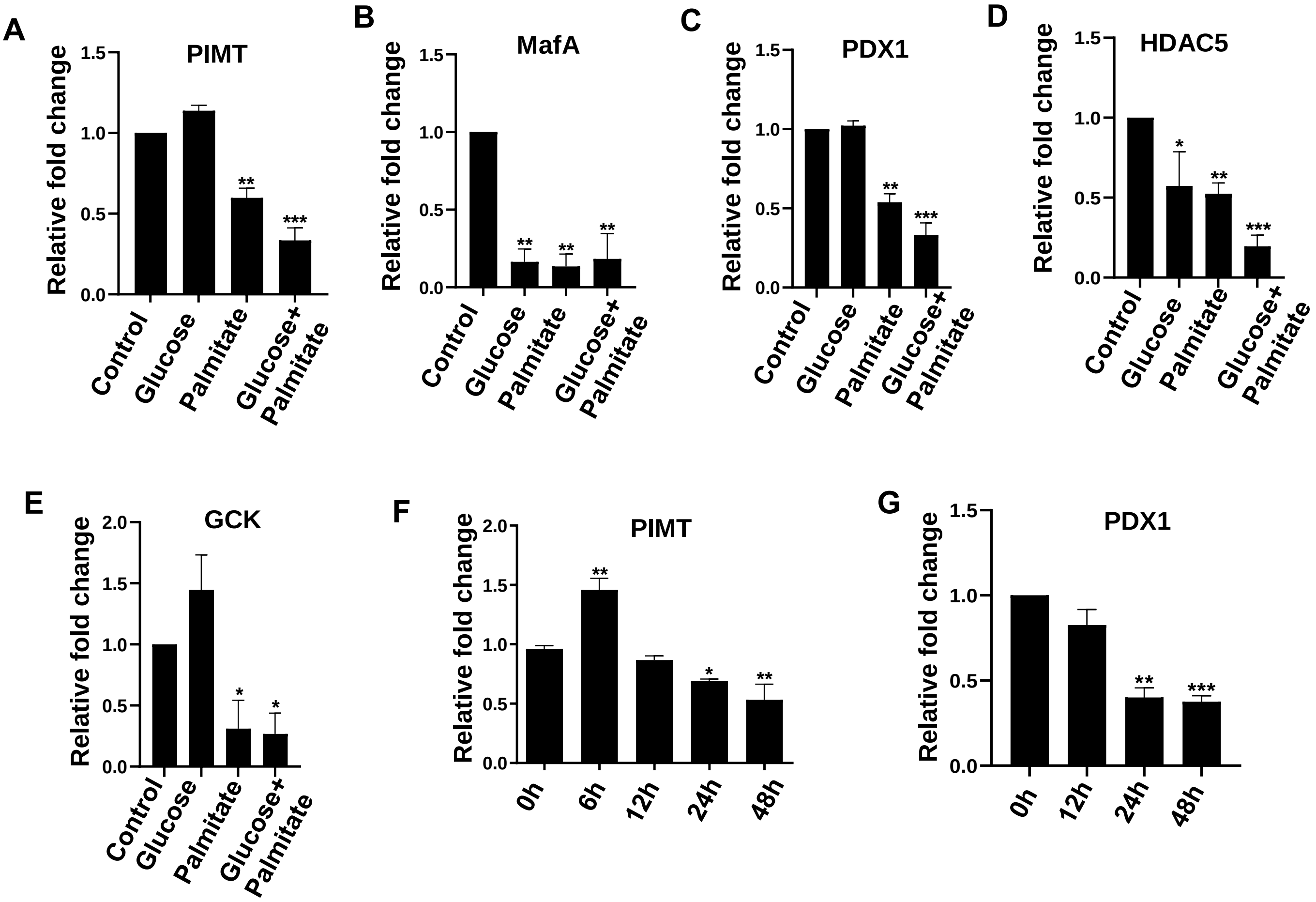

Figure S1. PIMT levels were decreased in BRIN-BD11 cells exposed to glucolipotoxicity and HFD-fed mice islets. A-E) Densitometric quantification of western blot shown in the Figure 1A. F) Densitometric quantification of PIMT western blot shown in the Figure 1G. G) Densitometric quantification of PDX1 western blot shown in the Figure 1H. Data are a representative of three independent experiments. Numerical data are shown as mean ± SD.

Statistical analysis was performed using Tukey's multiple comparison test. A *p* *< 0.05; **, *p*<0.01; ***, *p*<0.001 value was considered statistically significant.

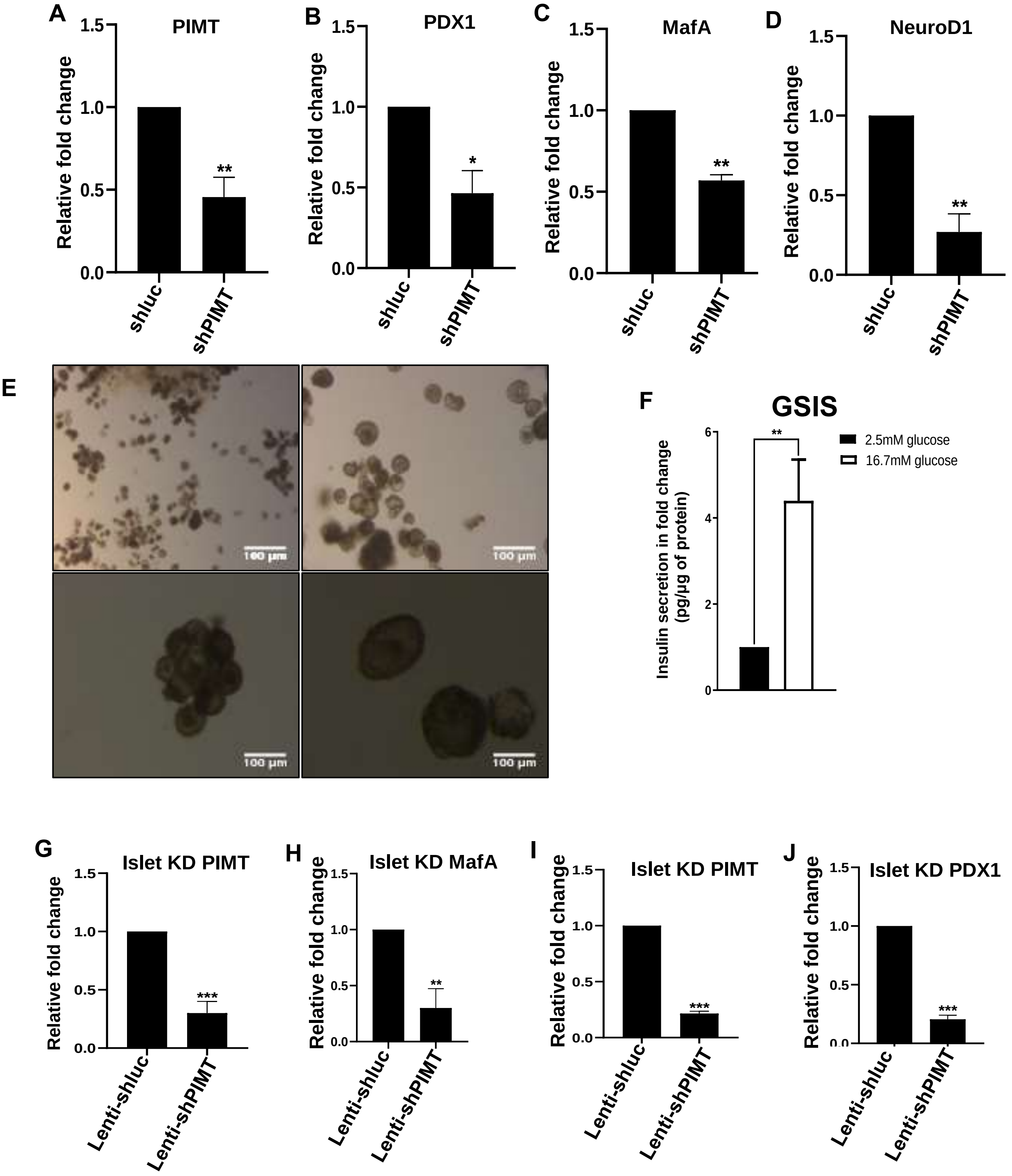

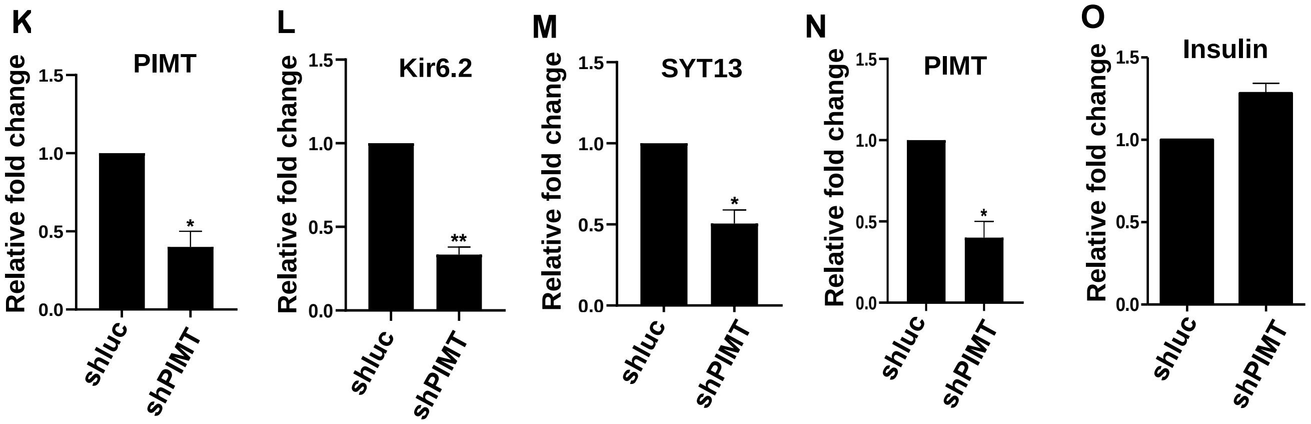

Figure S2. PIMT regulates Ins1 and Ins2 expression. A-D) Densitometric quantification of western blots shown in Figure 3G (A-C) and 3H (D). E, F) Microscopic images of isolated rat pancreatic islets using collagenase D (scale bar represents 100µM) and the associated glucose-stimulated insulin secretion analysis (F). G-M) Densitometric analysis of western blots shown in Figure 3I (G, H), 3J (I, J) and 3X (K-M). N-O) Densitometric analysis of western blots shown in Figure 3Z. Data are a representative of three independent experiments. Numerical data are shown as mean ± SD. Statistical analysis was performed using Student's t-test. A *p* *< 0.05; **, *p*<0.01; ***, *p*<0.001 value was considered statistically significant.

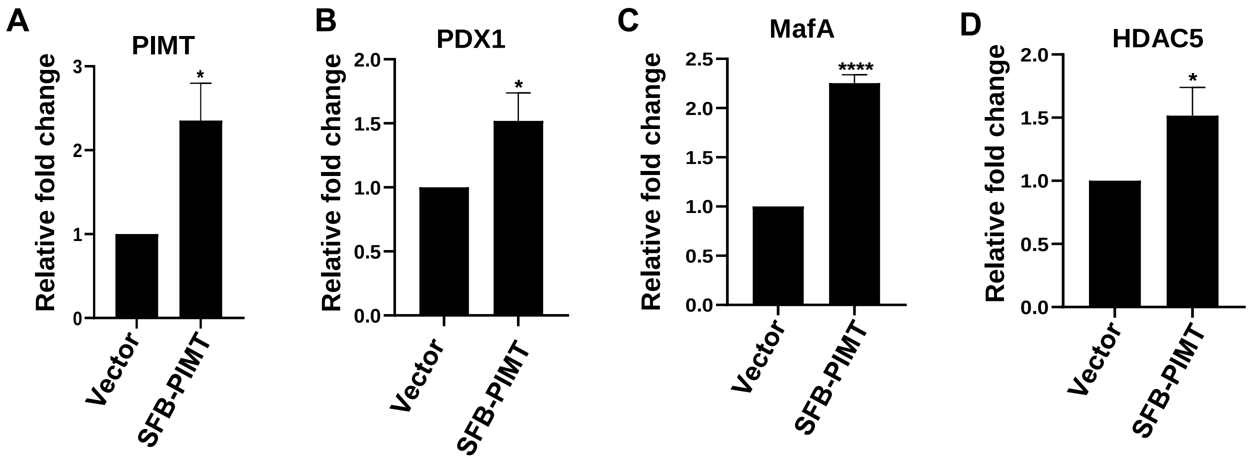

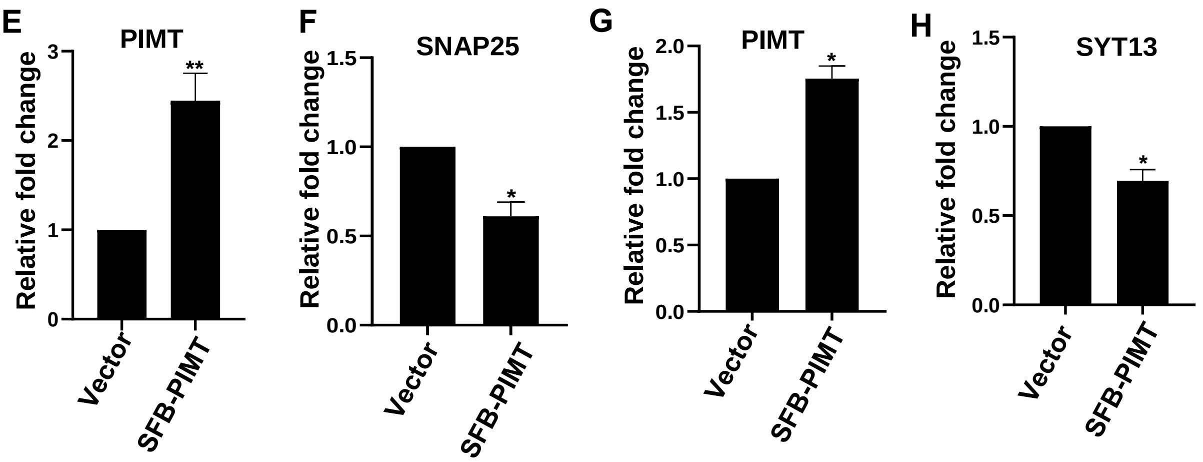

Figure S3. PIMT overexpression decreased the expression of key insulin secretory pathway genes. A-H) Densitometry quantification of western blots shown in Figure 6C (A-D), 6O (E,F) and 6P (G,H). Data are a representative of three independent experiments. Numerical data are shown as mean ± SD. Statistical analysis was performed using Student's t-test. A *p* *< 0.05; **, *p*<0.01; ****, *p*<0.0001 value was considered statistically significant.

Supplementary Table S1 (set 1 differentially expressed genes)

Ensemble ID	Gene name	p-value	log2FoldChange
ENSRNOG00000032699	Ttyh1	5.72682E-13	-1.665815526
ENSRNOG00000013330	Cdhr1	3.69444E-10	-0.980302709
ENSRNOG00000013564	Dok3	7.72787E-09	1.559647195
ENSRNOG00000005382	AABR07058423.1	1.96327E-08	3.678105511
ENSRNOG00000019685	Gdpd3	2.53408E-08	1.987673154
ENSRNOG00000012181	Lpl	4.70891E-08	-2.420951877
ENSRNOG00000027574	AABR07053879.1	1.34113E-07	1.319236566
ENSRNOG00000015421	Slc27a3	3.74955E-07	1.273897381
ENSRNOG00000026953	Gpr88	5.40818E-07	-1.05560244
ENSRNOG00000019422	Egr1	5.96187E-07	1.19209089
ENSRNOG00000004405	Pigr	1.45946E-06	-2.842641477
ENSRNOG00000011821	S100a4	3.15065E-06	1.836005578
ENSRNOG00000024470	Lman1	4.68397E-06	-0.784516959
ENSRNOG00000029260	Pitpnm2	5.17287E-06	-0.885400796
ENSRNOG00000032609	#N/A	6.24128E-06	1.929050009
ENSRNOG00000005498	Gcg	6.88895E-06	-2.597144206

ENSRNOG00000011517	Tnfrsf21	7.07569E-06	-1.497306792
ENSRNOG00000015825	Aurkc	9.47103E-06	0.980901161
ENSRNOG00000018029	Doc2g	9.64766E-06	0.955862002
ENSRNOG00000015860	Gipr	1.04376E-05	1.076596745
ENSRNOG00000002579	Parm1	1.39E-05	-0.856834044
ENSRNOG00000017206	Igfbp5	1.45529E-05	-1.150465478
ENSRNOG00000000500	Scube3	2.70983E-05	1.395204264
ENSRNOG00000060525	AABR07007717.3	2.80904E-05	-1.411416715
ENSRNOG00000008431	Gabbr2	2.99261E-05	-1.265404168
ENSRNOG00000032539	Spag8	3.26078E-05	1.279282439
ENSRNOG00000021602	Zmynd10	3.32921E-05	1.303475006
ENSRNOG00000043098	Mt2A	3.98829E-05	-2.479542342
ENSRNOG00000060063	Naa10	4.29984E-05	1.131488948
ENSRNOG00000050647	Hspa1b	6.34434E-05	-3.842803002
ENSRNOG00000034174	Bpifb4	6.76595E-05	1.113476607
ENSRNOG00000020957	Igflr1	6.86037E-05	1.358737115
ENSRNOG00000009967	Otof	7.96405E-05	1.075890772
ENSRNOG00000004273	Ifitm1	8.15392E-05	-1.449779336
ENSRNOG00000033262	Reep6	8.34184E-05	-0.890632849
ENSRNOG00000012237	Rdx	0.000103823	-0.911972654
ENSRNOG00000015696	Cdk5rap1	0.00010936	-0.995612473
ENSRNOG00000006646	Vopp1	0.000118338	-0.76788694
ENSRNOG00000014740	Gast	0.000126613	1.263111806
ENSRNOG00000033280	Pam	0.0001268	-0.754464837
ENSRNOG00000007044	L3mbtl1	0.0002192	1.444371326
ENSRNOG00000010938	Slc7a10	0.000301039	1.250846611
ENSRNOG00000029598	Robo2	0.000337949	-1.014697227
ENSRNOG00000047295	Prr22	0.000362729	0.99428091
ENSRNOG00000031333	#N/A	0.000380325	2.125939876
ENSRNOG00000042352	Dnase1l2	0.000444985	1.459025858
ENSRNOG00000003524	Lrrc59	0.000452949	-0.789308062
ENSRNOG00000054259	Klf11	0.000465331	-0.878277209
ENSRNOG00000020792	Etv4	0.000466234	1.258601975
ENSRNOG00000006280	Pcsk9	0.000479466	-1.551296633
ENSRNOG00000011526	Pcsk6	0.000488874	-1.008510604
ENSRNOG00000016216	Sft2d3	0.00049404	-0.863054404
ENSRNOG00000014795	Nr2f1	0.000506339	0.872851957
ENSRNOG00000058545	Arhgap4	0.000519885	0.899846728
ENSRNOG00000048172	Rac3	0.000544318	1.16916431
ENSRNOG00000046190	AC112062.1	0.000563085	2.693433381
ENSRNOG00000019830	Hsd17b1	0.000594279	0.839998069
ENSRNOG00000016244	Mical2	0.000598929	-0.757054585
ENSRNOG00000031915	Hdac10	0.000645102	0.834314548
ENSRNOG00000015085	Dmpk	0.000690341	0.829273814

ENSRNOG00000020148	Il4i1	0.000702059	0.947582135
ENSRNOG00000031852	Lsamp	0.000746342	1.378997119
ENSRNOG00000000439	Ager	0.000761431	1.224004455
ENSRNOG00000049077	Mir5125	0.00077681	1.037587775
ENSRNOG00000014215	Klf9	0.000834957	-1.077913625
ENSRNOG00000017914	Cavin3	0.00083682	-0.995996858
ENSRNOG00000018784	Jph3	0.000857404	-0.805349358
ENSRNOG00000000443	LOC103689965	0.000869946	1.495974771
ENSRNOG00000020342	Samd11	0.000902297	1.078784025
ENSRNOG00000025764	AC128848.1	0.000904035	-1.524240171
ENSRNOG00000021017	Ca11	0.000982514	0.806373667
ENSRNOG00000009514	Mme	0.000999543	-0.925979767
ENSRNOG00000017307	Prss23	0.001048088	-0.761426549
ENSRNOG00000008199	Odf3l2	0.001108107	1.234966713
ENSRNOG00000012757	Nek3	0.001140477	0.826211378
ENSRNOG00000060332	7SK	0.001181323	2.061395166
ENSRNOG00000018305	St8sia3	0.001183173	-0.869221123
ENSRNOG00000017045	Zdhhc1	0.001283508	0.986597844
ENSRNOG00000061908	7SK	0.001289834	2.90293685
ENSRNOG00000056643	Cdh8	0.001320817	0.910437516
ENSRNOG00000017899	Akr7a3	0.001413793	1.246854485
ENSRNOG00000038047	Mt1	0.001419188	-1.583652593
ENSRNOG00000040287	Cyp1b1	0.001502867	-1.496795525
ENSRNOG00000042601	Zglp1	0.001536011	1.40459739
ENSRNOG00000005413	Creb3l1	0.001653421	-0.861031945
ENSRNOG00000007077	Fem1b	0.001684731	-1.008261587
ENSRNOG00000018371	Tubb6	0.001742279	-0.799987499
ENSRNOG00000029465	Slc26a10	0.001758105	1.081116919
ENSRNOG00000018285	Kcna2	0.001771376	-0.766744146
ENSRNOG00000021016	Ntn5	0.001877823	1.196649825
ENSRNOG00000022844	Pdzrn4	0.001883244	-1.133740971
ENSRNOG00000025695	Tns3	0.001958362	-0.944013182
ENSRNOG00000006649	Thrb	0.002105953	-0.909423681
ENSRNOG00000037695	Sgpp2	0.002241219	-1.223311317
ENSRNOG00000061316	Ulbp1	0.002262593	-1.007486963
ENSRNOG00000053055	Otop3	0.002580042	-1.432770548
ENSRNOG00000043388	Irf3	0.002582309	0.851251797
ENSRNOG00000002950	Lyl1	0.00264858	0.955989736
ENSRNOG00000011411	Adgrg6	0.002711411	-1.412954812
ENSRNOG00000040205	Zcchc24	0.002730806	-1.347277562
ENSRNOG00000014297	Sdc4	0.002762165	-0.919432175
ENSRNOG00000047040	Lhb	0.002840011	0.859373781
ENSRNOG00000053909	7SK	0.002887878	2.25378016
ENSRNOG00000019662	Tm6sf1	0.00311086	-0.963011854

ENSRNOG00000017558	Tubb2a	0.003141438	-0.801929278
ENSRNOG00000015655	Ptgfrn	0.003239781	-0.741236568
ENSRNOG00000048230	LOC300308	0.003327132	0.872788836
ENSRNOG00000013658	Nefl	0.003466978	-0.975774126
ENSRNOG00000022505	Slc17a4	0.003491891	1.349432715
ENSRNOG00000003233	Serpinf2	0.003542785	1.083973863
ENSRNOG00000019065	Zfp385b	0.003621773	-1.074366731
ENSRNOG00000007445	Asph	0.003907965	-0.930319672
ENSRNOG00000043071	Linc00176	0.003929552	1.254554039
ENSRNOG00000019692	Metrn	0.004091536	1.814718356
ENSRNOG00000056565	AABR07059002.1	0.004110428	1.452207612
ENSRNOG00000025121	Pla2g3	0.004261341	0.808283613
ENSRNOG00000017508	Kmt5c	0.004527058	0.774661535
ENSRNOG00000011775	Mfap3l	0.004561431	-0.774118993
ENSRNOG00000017260	Cdr2	0.00461895	-0.880060429
ENSRNOG00000014232	P2ry1	0.004665784	-1.275298377
ENSRNOG00000056150	Purb	0.004694353	-0.805040659
ENSRNOG00000021689	Rps6kb2	0.004758589	0.783965029
ENSRNOG00000015541	Gnb3	0.004767901	0.871612707
ENSRNOG00000017117	Ybx2	0.004771149	0.870946687
ENSRNOG00000050860	Abcb11	0.004958455	1.303456902
ENSRNOG00000060123	Kifc2	0.005029679	1.046935974
ENSRNOG00000020505	Map4k1	0.005100664	0.770227061
ENSRNOG00000022699	AABR07030039.1	0.005189248	0.785547546
ENSRNOG00000049983	Shd	0.005313984	1.266220921
ENSRNOG00000013565	Zfp507	0.005542389	-1.026757687
ENSRNOG00000028744	Mtnr1a	0.005666454	-1.125985277
ENSRNOG00000009951	Aif1l	0.00574004	-0.814016523
ENSRNOG00000008626	Manea	0.005752139	-0.815563874
ENSRNOG00000055853	Gorasp2	0.005877558	-0.954877066
ENSRNOG00000001324	Asmt	0.005900009	0.951784089
ENSRNOG00000009103	Chrac1	0.005957186	-0.843880331
ENSRNOG00000013823	Cers3	0.006038291	-1.004310208
ENSRNOG00000013465	Tepp	0.00623285	1.101334338
ENSRNOG00000043311	Birc7	0.006244001	0.861120515
ENSRNOG00000028699	Sco1	0.006377486	-0.900580656
ENSRNOG00000004660	Fzd6	0.006455381	-0.899168981
ENSRNOG00000028235	RGD1560795	0.006458614	1.540650607
ENSRNOG00000004749	Slc30a1	0.00661486	-0.820999386
ENSRNOG00000021048	AC095693.1	0.006648502	1.236474638
ENSRNOG00000018715	Clec10a	0.006706208	-1.726834031
ENSRNOG00000025074	Fgg	0.00689073	-0.862324915
ENSRNOG00000015667	Epcam	0.006910679	-0.752977508
ENSRNOG00000010694	Tpbg	0.006929486	-0.89154727

ENSRNOG00000029778	Maob	0.007003647	-1.348820976
ENSRNOG00000003215	Zfp287	0.007158593	-0.813154041
ENSRNOG00000021714	Fam92b	0.007209091	1.100667495
ENSRNOG00000007151	Cdk14	0.007209524	-0.818427414
ENSRNOG00000018943	Tnnc1	0.007532678	0.833959506
ENSRNOG00000008843	Eci1	0.00768787	1.215052624
ENSRNOG00000010775	Arrdc4	0.008067884	-1.075220043
ENSRNOG00000047244	Nup214	0.008299735	-0.819430967
ENSRNOG00000020380	Lgals7	0.008459891	1.494564645
ENSRNOG00000016987	Pstpip2	0.008609826	-1.112894579
ENSRNOG00000005515	Rhbdl3	0.008891585	-0.766633675
ENSRNOG00000001711	Plaat1	0.009058187	-0.814969649
ENSRNOG00000007092	Fgb	0.009182422	-0.762514616
ENSRNOG00000009336	Mapk15	0.009420235	0.901740392
ENSRNOG00000010438	Cpt1b	0.009453324	0.788077525
ENSRNOG00000020770	Arl4d	0.009787533	0.944486246
ENSRNOG00000016526	Dsg2	0.009862964	-0.771193443
ENSRNOG00000047113	Spsb2	0.009956407	1.420680781
ENSRNOG00000006619	Dnajc9	0.00996089	-0.816108149
ENSRNOG00000003291	Creg1	0.01000572	-0.934020685
ENSRNOG00000008703	Rnps1	0.010237496	-0.904606385
ENSRNOG00000058604	AABR07012581.1	0.010274069	1.532210377
ENSRNOG00000011280	Mllt3	0.010318871	-0.833106469
ENSRNOG00000011964	Abcd4	0.010495538	0.799060304
ENSRNOG00000001785	Etv5	0.010510706	0.765578625
ENSRNOG00000047250	Gmfb	0.010588806	-0.79686872
ENSRNOG00000054542	#N/A	0.01068736	1.610597104
ENSRNOG00000032261	Cyp2d4	0.010862704	0.973654746
ENSRNOG00000000480	Phf1	0.011041712	0.831154883
ENSRNOG00000007489	Zfp41	0.011551213	-0.779876498
ENSRNOG00000059319	AABR07028013.1	0.0115682	1.077746517
ENSRNOG00000001520	Dlx1	0.011639922	-1.099193751
ENSRNOG00000043419	Hist3h2ba	0.011647808	0.801983246
ENSRNOG00000017893	Baiap3	0.011796892	1.679267223
ENSRNOG00000048186	F2rl3	0.011874458	1.03960159
ENSRNOG00000015075	Stc1	0.011930406	-1.3175173
ENSRNOG00000059962	AABR07025787.1	0.011950088	-1.106317163
ENSRNOG00000008482	Rbms1	0.0119759	-0.880606307
ENSRNOG00000003887	Lgi2	0.011997171	-0.869841678
ENSRNOG00000058260	Gpbar1	0.0121066	1.411862662
ENSRNOG00000003841	Kcnh1	0.012262876	-1.141695906
ENSRNOG00000053306	Washc1	0.012317274	0.801080856
ENSRNOG00000047401	Gfy	0.01233678	0.910665245
ENSRNOG00000010077	Smarcd3	0.01238971	0.816341179

ENSRNOG00000022796	Fgfbp3	0.012770244	-0.809911719
ENSRNOG00000053946	AC120807.1	0.012893492	1.135424667
ENSRNOG00000019771	Tbx6	0.013200117	1.220248755
ENSRNOG00000016214	Agl	0.013202361	-0.952232248
ENSRNOG00000028658	Olig2	0.013321827	-0.940304815
ENSRNOG00000050817	AABR07065113.1	0.013445947	0.805459543
ENSRNOG00000002292	Hnrnpd	0.013466724	-0.857978194
ENSRNOG00000013552	Scd	0.013622047	1.017168634
ENSRNOG00000025998	AABR07045485.1	0.013685666	0.936593531
ENSRNOG00000017220	Tcirg1	0.013820002	0.834726157
ENSRNOG00000016388	Sphkap	0.014039271	-1.132647411
ENSRNOG00000005917	Pawr	0.014266581	-0.787431239
ENSRNOG00000003840	Slit2	0.014407154	0.805273914
ENSRNOG00000004753	Napb	0.014483228	-1.435272513
ENSRNOG00000054978	H1f2	0.014588979	1.064157888
ENSRNOG00000060594	LOC100911672	0.014685036	0.920989084
ENSRNOG00000003127	Spryd4	0.014821093	-0.788853781
ENSRNOG00000014288	#N/A	0.015170915	-0.828933119
ENSRNOG00000042237	Tm6sf2	0.015172243	1.134883478
ENSRNOG00000009354	Nrarp	0.015633336	-0.84268071
ENSRNOG00000002642	Ptges3	0.015938206	-1.312310571
ENSRNOG00000010169	Atpaf1	0.015991115	-0.863203665
ENSRNOG00000014142	Ogfrl1	0.016261722	-1.059120592
ENSRNOG00000037225	Tyms	0.016271835	-0.785430161
ENSRNOG00000001053	Tmed2	0.016444109	-0.946286369
ENSRNOG00000016137	Rad23b	0.016741027	-0.783360472
ENSRNOG00000005615	Gadd45a	0.016873374	-0.763593476
ENSRNOG00000054322	#N/A	0.016990544	-2.350989015
ENSRNOG00000019723	LOC100910990	0.017095225	-1.045240532
ENSRNOG00000016273	Fam136a	0.017148103	-0.778187961
ENSRNOG00000009460	Pdzd8	0.017151835	-0.74969357
ENSRNOG00000027341	Tenm2	0.017247523	-0.841356936
ENSRNOG00000036667	Hexd	0.01737997	0.80395862
ENSRNOG00000006526	Sema3c	0.017485941	-1.133599102
ENSRNOG00000012502	Stk17b	0.017568581	-1.292001339
ENSRNOG00000062276	AABR07044631.2	0.01764689	1.577750946
ENSRNOG00000012215	Baiap2l2	0.018030221	1.088812413
ENSRNOG00000050414	AABR07006333.1	0.018141142	-1.170545553
ENSRNOG00000021155	Ctsk	0.018248826	1.253282902
ENSRNOG00000032254	Sin3a	0.018292897	-0.896978904
ENSRNOG00000022273	Rfk	0.018305094	-0.817183986
ENSRNOG00000049708	Wdr62	0.018422188	1.029592415
ENSRNOG00000010911	Tmem108	0.018499444	1.05334798
ENSRNOG00000010076	Pkp1	0.018651122	-0.933080498

ENSRNOG00000059903	Thbs3	0.01873886	0.945524232
ENSRNOG00000059714	Hsp90aa1	0.018868554	-0.87047932
ENSRNOG00000023433	Gata6	0.019197104	0.769714647
ENSRNOG00000013589	Cxcl12	0.019342552	0.949908829
ENSRNOG00000015701	Rreb1	0.019877243	-0.737978523
ENSRNOG00000012176	Rab27b	0.019881013	-0.931812884
ENSRNOG00000019240	Ampd2	0.020269504	0.964460732
ENSRNOG00000010947	Mmp14	0.020428901	-0.802427043
ENSRNOG00000017409	Wnt6	0.021020207	-1.442732093
ENSRNOG00000009528	Sdcbp2	0.021101166	1.081348048
ENSRNOG00000003908	Cep128	0.021209301	-0.797151365
ENSRNOG00000053143	AC119007.3	0.021356967	1.604624261
ENSRNOG00000012862	Spsb4	0.022257061	-0.780308778
ENSRNOG00000056247	#N/A	0.022258826	1.932393568
ENSRNOG00000013239	Tle4	0.022825447	-0.86467298
ENSRNOG00000055292	LOC679087	0.023216225	1.044823666
ENSRNOG00000020990	Fgf21	0.023241336	1.262423884
ENSRNOG00000016921	Klhl11	0.02352806	-0.771152647
ENSRNOG00000002810	Gfpt2	0.0239984	-0.829843453
ENSRNOG00000013057	Prc1	0.024040185	-0.875289995
ENSRNOG00000017900	rnf141	0.024209202	-0.978277868
ENSRNOG00000010584	Tmem123	0.024238863	-0.903334578
ENSRNOG00000005144	Tmem18	0.024330247	-0.798953736
ENSRNOG00000025644	LOC499331	0.024382378	-1.164469071
ENSRNOG00000017477	Mmp23	0.024393831	1.318647241
ENSRNOG00000053787	Mdfic	0.024400772	-1.049403162
ENSRNOG00000015068	Il11ra1	0.024429205	0.985534359
ENSRNOG00000059487	AABR07045405.1	0.024574525	-0.96395107
ENSRNOG00000046144	H3c1	0.024675628	1.068414355
ENSRNOG00000021318	Epas1	0.024722718	-0.739061119
ENSRNOG00000011560	Mtmr9	0.024845813	-0.747531568
ENSRNOG00000008526	Pdzd3	0.025030552	0.824988566
ENSRNOG00000026169	RGD1560146	0.025336592	1.665818093
ENSRNOG00000011987	Cd2ap	0.025446238	-0.927075705
ENSRNOG00000014183	Gnaq	0.025456311	-0.807657256
ENSRNOG00000061851	Foxj3	0.026095904	-1.05265835
ENSRNOG00000016309	Rgp1	0.026479094	-0.749178807
ENSRNOG00000023257	Adamts9	0.026695016	-0.822146174
ENSRNOG00000007374	Tac1	0.026936723	-1.239284877
ENSRNOG00000015615	Tnfrsf11a	0.026979677	-1.399128824
ENSRNOG00000006076	Steap2	0.0272796	-0.936025371
ENSRNOG00000016336	AABR07045373.1	0.027309559	0.961800932
ENSRNOG00000011794	Tcea3	0.027384816	1.098703134
ENSRNOG00000011854	Dennd11	0.027440696	-1.075538212

ENSRNOG00000000474	Rgl2	0.02766439	0.804371851
ENSRNOG00000018387	Wdr7	0.028263706	-0.861610933
ENSRNOG00000004791	Arf6	0.028534308	-0.778878529
ENSRNOG00000019128	St8sia4	0.028630128	-0.815337316
ENSRNOG00000057527	RNase_MRP	0.028700125	1.312966233
ENSRNOG00000003960	Cltrn	0.028800114	0.990010866
ENSRNOG00000019729	Jmjd8	0.028977976	0.910319898
ENSRNOG00000006395	#N/A	0.029233569	1.213539802
ENSRNOG00000008857	Adamts10	0.029270225	0.766136335
ENSRNOG00000017106	Csnk1a1	0.029382239	-0.78039335
ENSRNOG00000006148	RGD1310352	0.029383087	-0.893438665
ENSRNOG00000045646	LOC102549173	0.029466046	0.882905452
ENSRNOG00000027630	Casc1	0.02948609	0.774436717
ENSRNOG00000019096	Hsd17b3	0.030181145	-1.420687018
ENSRNOG00000043103	Frrs1l	0.030218506	-0.956380852
ENSRNOG00000032739	Da2-19	0.030229896	1.157631706
ENSRNOG00000018176	Rab6a	0.030454996	-0.883601889
ENSRNOG00000014613	Ddah1	0.030531954	-0.914494897
ENSRNOG00000029903	Spock3	0.030912891	0.833712057
ENSRNOG00000001510	AC120066.1	0.03093814	1.368724264
ENSRNOG00000010131	Rin2	0.031509177	-0.970615747
ENSRNOG00000046621	AABR07043748.1	0.031546778	-0.738711741
ENSRNOG00000046971	Ccer2	0.031627075	1.342721642
ENSRNOG00000009570	Pdxp	0.031665741	0.993377711
ENSRNOG00000000825	Calhm5	0.031721469	-0.87453675
ENSRNOG00000056193	AC108572.3	0.032047981	1.094631202
ENSRNOG00000055471	Ywhah	0.032057521	-0.978856232
ENSRNOG00000033411	Usp12	0.03211745	-0.737759823
ENSRNOG00000004104	Zkscan7	0.032388096	-0.765303931
ENSRNOG00000020460	#N/A	0.032450973	-0.834520185
ENSRNOG00000060141	Epdr1	0.032684936	-0.78207058
ENSRNOG00000014092	Paip2b	0.032766189	-1.253875684
ENSRNOG00000031790	H2bu1	0.032905605	1.152298799
ENSRNOG00000057556	Pdzrn3	0.032922949	-0.754247103
ENSRNOG00000036711	Spn	0.033233311	-0.787414526
ENSRNOG00000049056	AABR07051450.1	0.033364071	0.792646985
ENSRNOG00000036839	Hnrnpa1	0.033536047	-0.772923478
ENSRNOG00000013764	Fam118a	0.033584564	-0.805464731
ENSRNOG00000058484	Anp32a	0.033707806	-0.791492125
ENSRNOG00000023733	RGD1560821	0.033759352	0.816347664
ENSRNOG00000004730	Meis2	0.034029913	-0.935563067
ENSRNOG00000036918	Etfbkmt	0.034145751	-1.169990221
ENSRNOG00000053850	Rdh5	0.034270318	1.084117708
ENSRNOG00000021170	Plekho1	0.034493614	0.901822417

ENSRNOG00000016621	Actl7b	0.034610233	1.104218578
ENSRNOG00000009381	Mapk6	0.034682413	-0.928941417
ENSRNOG00000042753	Fgf13	0.034903753	-1.180590867
ENSRNOG00000050214	Amt	0.035159628	1.090750673
ENSRNOG00000018478	Myo3a	0.035234437	0.912050183
ENSRNOG00000024363	Sertad1	0.035261707	0.81638037
ENSRNOG00000008364	Cat	0.035764169	-1.13906523
ENSRNOG00000061925	AABR07059159.1	0.035871597	-1.172663747
ENSRNOG00000032224	RGD1562378	0.035931175	1.283637524
ENSRNOG00000052051	Slc24a1	0.036091091	-1.234859696
ENSRNOG00000046794	Khdrbs1	0.036927125	-0.844176226
ENSRNOG00000016330	Rbm12b	0.036976316	1.326877185
ENSRNOG00000028422	Rmnd5a	0.037112439	-1.070339426
ENSRNOG00000009175	Jagn1	0.037256896	-0.79764181
ENSRNOG00000007088	Xpot	0.037292575	-0.836623327
ENSRNOG00000001890	Txnrd2	0.037642106	0.778956368
ENSRNOG00000016459	Eif3j	0.037794665	-1.01618198
ENSRNOG00000033912	Fcho1	0.037797874	0.855199613
ENSRNOG00000057966	Smim31	0.037856531	-0.943191055
ENSRNOG00000045749	AC105515.1	0.038135595	1.145945602
ENSRNOG00000007755	Pm20d2	0.038314409	-1.407408487
ENSRNOG00000004284	Btg1	0.038618367	-0.969447078
ENSRNOG00000031669	Lpp	0.03878304	1.172180261
ENSRNOG00000016260	Polr3g	0.03976816	-0.868864926
ENSRNOG00000012311	Slc35d3	0.03985226	-0.837881014
ENSRNOG00000003400	Fmo4	0.039893734	-1.17677829
ENSRNOG00000043233	Cyp4f6	0.040041982	-1.00135535
ENSRNOG00000004292	Sptssa	0.040061473	-0.854970254
ENSRNOG00000014647	Cbfb	0.040267867	-1.254662974
ENSRNOG00000034228	Pik3c2g	0.04061542	-1.29574534
ENSRNOG00000060349	Fam3c	0.041734892	-0.771516035
ENSRNOG00000053679	AABR07060862.1	0.042364949	-1.450376056
ENSRNOG00000020106	Tnfrsf4	0.042391949	1.054680588
ENSRNOG00000001431	Rasa4	0.042636996	0.983808446
ENSRNOG00000037647	Ostm1	0.042680605	-0.778269909
ENSRNOG00000017751	Arl6ip1	0.043130245	-1.021109732
ENSRNOG00000021263	Tmem230	0.043530979	-0.817243644
ENSRNOG00000057451	Itga5	0.043577153	-0.774982472
ENSRNOG00000050289	Cstf2t	0.043778153	-0.77594947
ENSRNOG00000004874	Flrt3	0.043906517	-1.128959723
ENSRNOG00000051650	Ywhaq	0.044154126	-0.846000759
ENSRNOG00000026527	#N/A	0.044283429	0.910465701
ENSRNOG00000014964	Hp	0.044452159	0.938251719
ENSRNOG00000020389	Capn12	0.044554012	1.102292527

ENSRNOG00000033010	Akr1c12	0.044811158	-1.060850166
ENSRNOG00000055277	LOC100362333	0.044812358	-0.846530575
ENSRNOG00000011535	Gcsh	0.045694478	-0.905930204
ENSRNOG00000045698	Lin7c	0.045858604	-0.810930701
ENSRNOG00000017904	Nol7	0.045977143	-0.873583697
ENSRNOG00000019959	Kcnc3	0.046134529	0.777795415
ENSRNOG00000023320	#N/A	0.046213147	0.861002155
ENSRNOG00000042374	Tgif2	0.046404694	0.997980084
ENSRNOG00000059572	Tsen34l1	0.046479714	-1.545335251
ENSRNOG00000011861	Aadat	0.046875879	-0.90069536
ENSRNOG00000020723	Pten	0.047172096	-0.810113015
ENSRNOG00000003247	Sde2	0.04745819	-0.947385754
ENSRNOG00000024595	Cers6	0.047583553	-0.777002563
ENSRNOG00000024757	AABR07069219.1	0.047634438	-0.909734103
ENSRNOG00000028576	AABR07028446.1	0.047656054	0.888606044
ENSRNOG00000052421	AABR07012475.2	0.047685324	0.789626429
ENSRNOG00000013670	Pax3	0.047753483	-1.421393461
ENSRNOG00000018770	Pmaip1	0.047803184	-1.081886285
ENSRNOG00000020927	Tulp2	0.048091739	1.273362887
ENSRNOG00000047895	Mettl1	0.048285147	-1.091981749
ENSRNOG00000005285	Fbxo33	0.049665384	-0.927689394
ENSRNOG00000014899	AC110824.1	0.049921147	-1.498771364

Supplementary Table S2 (set 2 differentially expressed genes)

Ensemble ID	Gene name	p-value	log2FoldChange
ENSRNOG00000002413	Gpc4	4.18674E-37	1.991742964
ENSRNOG00000000825	Calhm5	2.03493E-27	3.973986669
ENSRNOG00000037188	Mug1	2.31596E-24	4.665979596
ENSRNOG00000045919	LOC103694120	7.28286E-21	5.503619366
ENSRNOG00000013092	Lonrf3	4.24698E-20	1.062354276
ENSRNOG00000012906	Bcas1	2.98693E-19	-4.061333043
ENSRNOG00000024763	LOC685431	5.19913E-19	1.249990248
ENSRNOG00000057527	RNase_MRP	1.6798E-18	2.145288629
ENSRNOG00000018229	Slc45a1	2.19575E-18	-3.453445388
ENSRNOG00000051483	LOC103689961	1.3678E-17	1.467227778
ENSRNOG00000061230	L1cam	1.68977E-17	-0.93104142
ENSRNOG00000020325	Calhm2	4.57156E-17	1.699768133
ENSRNOG00000006069	Eif2ak3	1.4212E-16	-1.050123237

ENSRNOG00000016541	Enc1	4.04918E-16	-0.913724251
ENSRNOG00000047401	Gfy	1.85951E-15	1.945858973
ENSRNOG00000003769	Tmem163	1.67544E-14	1.043779198
ENSRNOG00000008837	Ass1	5.04676E-14	0.839209015
ENSRNOG00000043419	Hist3h2ba	7.54629E-14	1.090622996
ENSRNOG00000017403	Apobr	8.86656E-14	-2.404044613
ENSRNOG00000013916	Nefm	1.05679E-13	-1.294533803
ENSRNOG00000015593	Sigirr	2.38785E-13	0.875016443
ENSRNOG00000018420	Slc22a7	6.2352E-13	1.816007548
ENSRNOG00000014387	Chac1	7.32434E-13	-1.235760366
ENSRNOG00000000878	Slc44a4	7.36685E-13	1.047562435
ENSRNOG00000009031	Gucy2c	3.40896E-12	2.379194632
ENSRNOG00000019318	Syt3	1.2883E-11	1.262692253
ENSRNOG00000020151	Cdh1	1.37095E-11	1.230124167
ENSRNOG00000015860	Gipr	2.09219E-11	1.27997949
ENSRNOG00000054259	Klf11	3.02709E-11	-0.917846899
ENSRNOG00000024712	Insc	3.8602E-11	-2.145929455
ENSRNOG00000005248	Slc1a4	4.20647E-11	-0.90437716
ENSRNOG00000026605	Ifi27l2b	4.43352E-11	-1.377519482
ENSRNOG00000046404	LOC291276	5.71481E-11	3.718902121
ENSRNOG00000022337	Slitrk6	9.62301E-11	-1.103912414
ENSRNOG00000001074	Mphosph9	1.14208E-10	-0.984642311
ENSRNOG00000030118	Msn	1.38595E-10	-1.073210303
ENSRNOG00000012052	Ins1	1.70908E-10	1.759232996
ENSRNOG00000007713	Tmcc3	1.9019E-10	-0.812578557
ENSRNOG00000001187	Oasl	3.48112E-10	-1.000234249
ENSRNOG00000019685	Gdpd3	4.64619E-10	2.814564113
ENSRNOG00000004692	A1bg	6.11901E-10	1.466578899
ENSRNOG00000012181	Lpl	7.28517E-10	-1.892547537
ENSRNOG00000008478	Mmp13	7.58615E-10	3.051167851
ENSRNOG00000011032	Lhfpl2	1.1671E-09	-0.915620684
ENSRNOG00000000723	RT1-CE5	1.44451E-09	0.944684385
ENSRNOG00000025702	Kdm2b	1.60212E-09	-0.95646682
ENSRNOG00000020380	Lgals7	1.77812E-09	2.297709636
ENSRNOG00000013851	Spry4	3.02003E-09	-0.980601063
ENSRNOG00000028713	Acvrl1	3.90711E-09	1.693673026
ENSRNOG00000049349	Tafa5	6.494E-09	-1.226374407
ENSRNOG00000020505	Map4k1	8.60295E-09	0.913202148
ENSRNOG00000055672	Gpx2	9.42718E-09	-3.451645872
ENSRNOG00000020583	Fcgrt	1.26819E-08	1.772832652
ENSRNOG00000008536	Actc1	1.43617E-08	-2.40418825
ENSRNOG00000020531	Fen1	2.29769E-08	-0.811675199
ENSRNOG00000032590	Ttc28	3.99255E-08	-1.185204316
ENSRNOG00000021102	Scn1b	4.02643E-08	1.58475123

ENSRNOG00000028235	RGD1560795	4.6759E-08	1.500837224
ENSRNOG00000028543	AABR07052523.1	1.15755E-07	1.095463428
ENSRNOG00000025055	Zdhhc19	1.3951E-07	-2.411639305
ENSRNOG00000060568	Ptpn21	1.51535E-07	-0.81526306
ENSRNOG00000001520	Dlx1	1.75366E-07	-1.128945337
ENSRNOG00000013658	Nefl	1.76964E-07	-1.137446144
ENSRNOG00000031540	LOC100362384	1.83496E-07	0.877224192
ENSRNOG00000012862	Spsb4	1.99228E-07	-1.025345036
ENSRNOG00000022699	AABR07030039.1	3.22446E-07	1.113944525
ENSRNOG00000024517	Gpr119	3.45567E-07	1.60089125
ENSRNOG00000000699	Selplg	3.95946E-07	3.65338239
ENSRNOG00000019500	Cyp1a1	4.02631E-07	-1.831788643
ENSRNOG00000011541	Cygb	4.12137E-07	-0.781686982
ENSRNOG00000042860	Pappa2	4.13358E-07	0.977528215
ENSRNOG00000027767	Slc38a5	4.35948E-07	1.353652003
ENSRNOG00000016695	Mmp2	4.45573E-07	-1.159923504
ENSRNOG00000014550	Plcxd3	4.64444E-07	-1.304584777
ENSRNOG00000024093	Dmrta1	7.25696E-07	-0.926326628
ENSRNOG00000000925	Psph	1.32527E-06	-0.795667907
ENSRNOG00000042980	Adam19	1.34748E-06	-1.477887233
ENSRNOG00000031930	Bin2	1.61813E-06	-1.908535769
ENSRNOG00000013605	AC120568.1	2.02953E-06	1.920251733
ENSRNOG00000049269	RGD1563294	2.07176E-06	0.984726204
ENSRNOG00000028744	Mtnr1a	2.25085E-06	-1.188127071
ENSRNOG00000046007	Cldn3	2.42622E-06	0.917292051
ENSRNOG00000017072	Slc16a14	2.42968E-06	-1.104381924
ENSRNOG00000023458	Dcaf12l1	2.62086E-06	-1.030529193
ENSRNOG00000053055	Otop3	2.70252E-06	2.34073544
ENSRNOG00000046428	Lrrc75b	2.77975E-06	-0.739282859
ENSRNOG00000033722	Rnf207	3.42233E-06	-1.701774042
ENSRNOG00000003807	Wnt9b	3.96513E-06	-1.748367489
ENSRNOG00000011821	S100a4	3.97527E-06	1.187036503
ENSRNOG00000006855	Padi4	5.20669E-06	1.848846018
ENSRNOG00000018285	Kcna2	5.83E-06	-0.804578376
ENSRNOG00000054246	LOC679711	6.0546E-06	1.145466372
ENSRNOG00000020259	RGD1305347	6.12461E-06	1.12706301
ENSRNOG00000014740	Gast	6.76595E-06	1.111344935
ENSRNOG00000022921	Dact2	7.9998E-06	0.787650963
ENSRNOG00000004500	Myc	8.43466E-06	1.634493803
ENSRNOG00000017525	Epha1	8.70549E-06	0.959502409
ENSRNOG00000037167	Rtp3	8.75224E-06	-1.292868907
ENSRNOG00000032609	AY172581.18	9.24167E-06	1.778449346
ENSRNOG00000052368	Clcnka	9.76069E-06	1.191707463
ENSRNOG00000007490	Gabrr2	9.82101E-06	-3.087354549

ENSRNOG00000007489	Zfp41	1.16563E-05	-0.763037026
ENSRNOG00000008487	Amotl2	1.52967E-05	-1.623301894
ENSRNOG00000027030	Adm	1.53782E-05	1.035522848
ENSRNOG00000009577	Ndst4	1.56483E-05	-1.576188652
ENSRNOG00000052667	AABR07027555.1	1.57117E-05	1.230511798
ENSRNOG00000000873	Brs3	1.57656E-05	-1.701585639
ENSRNOG00000020517	Prrg2	1.78797E-05	0.768548172
ENSRNOG00000003832	Vash2	2.01673E-05	-0.747508731
ENSRNOG00000001151	Sirt4	2.09198E-05	0.768534846
ENSRNOG00000016244	Mical2	2.15672E-05	-1.157272822
ENSRNOG00000012237	Rdx	2.44548E-05	-1.064944653
ENSRNOG00000027574	AABR07053879.1	2.56088E-05	1.699493504
ENSRNOG00000026212	Micall1	2.65156E-05	-0.73984492
ENSRNOG00000014610	Anpep	2.67044E-05	0.77619217
ENSRNOG00000020792	Etv4	2.6822E-05	-1.0068985
ENSRNOG00000021318	Epas1	2.73156E-05	-0.764069594
ENSRNOG00000001191	Tchp	3.01154E-05	-0.740792153
ENSRNOG00000047712	Dcx	3.32393E-05	-2.225181393
ENSRNOG00000025624	Arhgap20	3.55607E-05	-0.843080296
ENSRNOG00000040205	Zcchc24	4.42886E-05	-1.507262732
ENSRNOG00000013304	Arg1	4.71E-05	-1.258458273
ENSRNOG00000010011	Osbpl3	5.06829E-05	-1.122727316
ENSRNOG00000054978	H1f2	5.41898E-05	1.377826537
ENSRNOG00000005382	AABR07058423.1	5.65059E-05	1.517212553
ENSRNOG00000021802	Isg15	6.12576E-05	-1.194273144
ENSRNOG00000016299	Klf4	6.15989E-05	-0.994085318
ENSRNOG00000012721	Ednra	6.20673E-05	-1.5049246
ENSRNOG00000006526	Sema3c	6.34593E-05	-0.79552083
ENSRNOG00000007607	Nr4a1	6.75216E-05	0.869054075
ENSRNOG00000025354	Rinl	6.77615E-05	0.813853835
ENSRNOG00000003870	C1qtnf2	6.86041E-05	1.318945948
ENSRNOG00000048927	AABR07018533.1	7.3982E-05	0.865836472
ENSRNOG00000049334	Atp2c2	7.52667E-05	1.040672823
ENSRNOG00000013209	Barhl1	7.58384E-05	0.939611526
ENSRNOG00000031852	Lsamp	8.24901E-05	1.138370716
ENSRNOG00000004709	Foxn3	8.84845E-05	-0.875234997
ENSRNOG00000007151	Cdk14	8.86267E-05	-0.874423908
ENSRNOG00000025644	LOC499331	9.12598E-05	-0.790286767
ENSRNOG00000007951	Galnt14	9.38337E-05	0.910370611
ENSRNOG00000013166	Wnt4	9.65255E-05	1.471465804
ENSRNOG00000015550	Ptgds	9.77039E-05	-1.12949946
ENSRNOG00000054563	Selenoh	0.000103269	0.883856225
ENSRNOG00000026169	RGD1560146	0.000104151	4.640830191
ENSRNOG00000059362	Has3	0.000105092	-0.877358178

ENSRNOG00000056247	7SK	0.000107635	1.667068937
ENSRNOG00000012345	AABR07004276.1	0.000123367	1.165329757
ENSRNOG00000034106	AC127887.1	0.000124203	0.938904941
ENSRNOG00000031333	AY172581.10	0.000124903	1.865353802
ENSRNOG00000014746	Dzip1l	0.000130392	-1.938488478
ENSRNOG00000049402	Nbl1	0.000148075	1.343702751
ENSRNOG00000013102	Entpd2	0.000162826	0.775546312
ENSRNOG00000013196	Dok5	0.000169149	1.027765014
ENSRNOG00000020465	Ripk3	0.000169223	1.829236622
ENSRNOG00000049105	AC095947.3	0.000172867	0.79738855
ENSRNOG00000003251	B3galt2	0.000174032	1.200083829
ENSRNOG00000013239	Tle4	0.000184497	-0.850683713
ENSRNOG00000059373	LOC100909548	0.000193909	0.840806062
ENSRNOG00000018301	AABR07039356.1	0.000205999	1.162147068
ENSRNOG00000014233	Krt19	0.000213588	1.860578051
ENSRNOG00000004317	Vipr2	0.000264344	-0.833027713
ENSRNOG00000013090	Gadd45g	0.000271022	1.106071693
ENSRNOG00000006144	Cntn3	0.000274295	1.026010741
ENSRNOG00000006860	Itk	0.000295791	-1.316655801
ENSRNOG00000033490	Vsig2	0.000296624	1.415987606
ENSRNOG00000012176	Rab27b	0.000299205	-1.035847945
ENSRNOG00000033613	AABR07030647.1	0.000302775	1.300140437
ENSRNOG00000033090	Ltbp1	0.00030564	-0.81649868
ENSRNOG00000056343	Lats2	0.000306253	-0.945852539
ENSRNOG00000060332	7SK	0.000323146	1.797437729
ENSRNOG00000060525	AABR07007717.3	0.000323665	-1.188076557
ENSRNOG00000006170	Bach2	0.000330684	-0.834517731
ENSRNOG00000053334	Stmn4	0.000338217	-1.035763713
ENSRNOG00000003654	Cldn9	0.000344622	1.082449805
ENSRNOG00000046190	AC112062.1	0.000349387	1.751999366
ENSRNOG00000049162	Prr15l	0.000351627	1.542303719
ENSRNOG00000021259	Prnp	0.000361008	-0.875670655
ENSRNOG00000029543	Cish	0.000370379	0.820059985
ENSRNOG00000020587	Efemp2	0.000393534	1.075042012
ENSRNOG00000019723	LOC100910990	0.00040886	-1.027883276
ENSRNOG00000014265	Tnfrsf19	0.000414411	-0.819711982
ENSRNOG00000010983	Otog	0.000416347	2.181782092
ENSRNOG00000004730	Meis2	0.00042577	-1.158979972
ENSRNOG00000019422	Egr1	0.000430006	0.811199226
ENSRNOG00000004192	Arhgap30	0.000430255	1.64127311
ENSRNOG00000000055	Fcrl6	0.000461294	-2.601186306
ENSRNOG00000007462	Septin8	0.000503161	-0.831961024
ENSRNOG00000050933	LOC100912564	0.000516318	1.444687566
ENSRNOG00000043193	Smim1	0.000525795	2.102829852

ENSRNOG00000016885	Klf6	0.000532182	-1.06556704
ENSRNOG00000032224	RGD1562378	0.00053231	1.555975892
ENSRNOG00000032539	Spag8	0.000533373	1.045046709
ENSRNOG00000037196	Spag17	0.000593101	-1.370394202
ENSRNOG00000033688	Cep170b	0.000614144	-0.772407483
ENSRNOG00000005515	Rhbdl3	0.00063466	-0.775522586
ENSRNOG00000059319	AABR07028013.1	0.000638843	0.946030901
ENSRNOG00000029598	Robo2	0.000673221	-0.790176824
ENSRNOG00000023733	RGD1560821	0.000683775	1.230173106
ENSRNOG00000032240	Gbp5	0.000716793	-1.65630239
ENSRNOG00000022256	Cxcl10	0.00072023	-1.129024711
ENSRNOG00000012807	C1qa	0.00072614	1.411085214
ENSRNOG00000030712	RT1-A2	0.000742162	1.212321637
ENSRNOG00000020830	LOC108348128	0.000747846	1.339263307
ENSRNOG00000004854	Has2	0.000826602	-1.567621509
ENSRNOG00000057556	Pdzrn3	0.000839651	-1.188135411
ENSRNOG00000033734	Tnnt2	0.00091725	1.550065508
ENSRNOG00000014171	Tnfsf13	0.000979073	-1.059464344
ENSRNOG00000028576	AABR07028446.1	0.001005162	-1.146023063
ENSRNOG00000024433	Fbxl7	0.001038062	-1.073327167
ENSRNOG00000029115	LOC102555453	0.001049966	0.8042756
ENSRNOG00000013465	Tepp	0.001053818	0.799771695
ENSRNOG00000057207	AABR07014239.1	0.001105522	2.099017624
ENSRNOG00000006383	Atoh1	0.001107682	-1.2030408
ENSRNOG00000051957	AC126897.1	0.001112112	0.814283251
ENSRNOG00000000728	Clic2	0.001193714	2.349714288
ENSRNOG00000029082	AABR07005031.1	0.001228285	0.889639478
ENSRNOG00000003800	Rgs9	0.001302687	0.821041527
ENSRNOG00000039744	RT1-CE4	0.001333341	0.889386549
ENSRNOG00000059041	AC141169.5	0.001350795	0.962857165
ENSRNOG00000053909	7SK	0.001351869	1.831664784
ENSRNOG00000003841	Kcnh1	0.001487189	-1.132246209
ENSRNOG00000004319	AABR07038019.1	0.00150484	0.869583317
ENSRNOG00000019560	Pde2a	0.001509998	0.904648031
ENSRNOG00000058507	AABR07041972.1	0.0015497	1.536666513
ENSRNOG00000027506	Zp4	0.001597727	-1.30862997
ENSRNOG00000014083	Iqsec3	0.001629071	-0.838979277
ENSRNOG00000025164	Bhlha15	0.001714838	1.098381351
ENSRNOG00000019129	Fcgbp	0.001737149	1.195879503
ENSRNOG00000016309	Rgp1	0.001859612	-0.76376732
ENSRNOG00000053787	Mdfic	0.0019277	-0.940846161
ENSRNOG00000056863	AABR07050116.1	0.002004692	1.348982297
ENSRNOG00000007964	Tp53inp1	0.002121726	-0.911920074
ENSRNOG00000046790	AABR07040887.1	0.002137433	-0.860960999

ENSRNOG00000005391	Prex2	0.00213918	-0.924146427
ENSRNOG00000013213	Epha4	0.002143616	-0.811907739
ENSRNOG00000048704	AABR07032392.1	0.002156697	0.921405137
ENSRNOG00000008979	Guca2b	0.002228311	-1.642294972
ENSRNOG00000007574	Padi2	0.00226454	0.931100681
ENSRNOG00000000574	AC096404.1	0.002279257	1.026224643
ENSRNOG00000045998	Sema6b	0.002287016	-0.952922253
ENSRNOG00000022555	LOC497899	0.002304628	0.816816003
ENSRNOG00000055962	Bgn	0.002395921	-1.335338606
ENSRNOG00000048758	Snrpd2l	0.002407902	0.77435385
ENSRNOG00000053946	AC120807.1	0.002415072	1.055137871
ENSRNOG00000017063	Fcna	0.002423957	1.06732856
ENSRNOG00000012417	Iapp	0.002450173	1.166679394
ENSRNOG00000013971	Psat1	0.002453296	-1.071519686
ENSRNOG00000003891	Dio2	0.002454257	-1.049702633
ENSRNOG00000015971	Slc12a2	0.002486844	-0.75379729
ENSRNOG00000003496	Tbc1d9	0.002589181	-0.913080269
ENSRNOG00000049983	Shd	0.002704638	1.176268558
ENSRNOG00000005284	Itpka	0.002840751	1.006394813
ENSRNOG00000004089	Enpp2	0.002890282	0.972397159
ENSRNOG00000009892	Adamts15	0.002937411	-1.419729879
ENSRNOG00000052564	Gpx3	0.002984019	0.953206709
ENSRNOG00000029260	Pitpnm2	0.003001435	-1.014623339
ENSRNOG00000036960	Abcc9	0.003008023	-1.483293018
ENSRNOG00000049277	Syne4	0.003078	1.188009956
ENSRNOG00000011250	Inmt	0.003191062	-1.008037622
ENSRNOG00000014232	P2ry1	0.003258394	-1.094865973
ENSRNOG00000027350	Tns4	0.003269667	0.96728187
ENSRNOG00000023257	Adamts9	0.003281507	-0.802931399
ENSRNOG00000006649	Thrb	0.003304354	-0.742227164
ENSRNOG00000018254	Sncaip	0.003334554	-1.169963541
ENSRNOG00000015618	Wnt5a	0.003336455	-0.946281238
ENSRNOG00000019050	Ifit1	0.003346389	-1.911970648
ENSRNOG00000003736	Col5a2	0.003369039	-1.352821816
ENSRNOG00000055755	AABR07030086.1	0.003387487	0.862223925
ENSRNOG00000046905	Sgce	0.003496687	1.607889801
ENSRNOG00000033575	Emid1	0.003612133	-0.759135707
ENSRNOG00000017800	Foxc1	0.003621568	-0.92160182
ENSRNOG00000009204	Il17re	0.003679556	-1.170617813
ENSRNOG00000013565	Zfp507	0.003764378	-0.768584964
ENSRNOG00000007539	Rsad2	0.003818016	-1.060314531
ENSRNOG00000015696	Cdk5rap1	0.003892158	-0.814934377
ENSRNOG00000001618	Ripk4	0.003892788	-0.831678883
ENSRNOG00000021507	Luzp2	0.003924736	1.371973884

ENSRNOG00000005690	Lmcd1	0.004288685	-1.504622026
ENSRNOG00000003143	RGD1310166	0.004352383	1.373008498
ENSRNOG00000046261	Acp5	0.004367412	0.841711532
ENSRNOG00000050478	LOC102548682	0.004398414	1.138397838
ENSRNOG00000001474	Tmem270	0.004541764	-0.897775968
ENSRNOG00000030296	AABR07071195.1	0.004563198	1.17424341
ENSRNOG00000000463	Col11a2	0.004612014	-1.077577862
ENSRNOG00000002802	Cxcl1	0.004619942	-1.45215074
ENSRNOG00000025074	Fgg	0.004635622	1.307569121
ENSRNOG00000051252	AABR07038873.1	0.004705134	0.830089729
ENSRNOG00000005041	Crip2	0.004760971	-0.845584889
ENSRNOG00000025332	Cd109	0.004786794	-1.321509882
ENSRNOG00000059927	AABR07044414.1	0.004978074	1.376148399
ENSRNOG00000009528	Sdcbp2	0.004979644	0.981363292
ENSRNOG00000046022	Wdr77	0.005037347	1.020052356
ENSRNOG00000053516	AABR07044454.1	0.005097454	-1.631193614
ENSRNOG00000005673	Runx1t1	0.005804249	-1.461115402
ENSRNOG00000033528	Tll1	0.005820792	-0.76367317
ENSRNOG00000020112	AABR07036567.1	0.005860278	1.075513536
ENSRNOG00000017319	Mertk	0.006096824	-0.772349519
ENSRNOG00000060442	AC091481.1	0.006103321	-0.775805943
ENSRNOG00000015075	Stc1	0.006163495	1.161418768
ENSRNOG00000058555	7SK	0.006193733	1.386918666
ENSRNOG00000048951	LOC100364500	0.00645321	1.311427421
ENSRNOG00000008703	Rnps1	0.006491242	-0.760554747
ENSRNOG00000028266	Lrrc55	0.00665045	-0.909077365
ENSRNOG00000004794	Rtn1	0.006677015	-0.812029442
ENSRNOG00000018778	Cadm1	0.006717521	-1.104627111
ENSRNOG00000054890	Flna	0.006721398	-0.811721181
ENSRNOG00000042101	Zfp93	0.006742485	0.862037329
ENSRNOG00000023917	AABR07034362.1	0.006836	0.973981465
ENSRNOG00000007197	Nr1h4	0.006892247	-0.864236971
ENSRNOG00000042787	Fam229a	0.007016731	0.877441643
ENSRNOG00000038864	AC135409.1	0.007073802	0.791543758
ENSRNOG00000004482	Ccdc88c	0.007525808	-0.776965992
ENSRNOG00000027066	Stk32a	0.007966846	-0.830758437
ENSRNOG00000062225	AABR07002792.2	0.007970422	0.918781345
ENSRNOG00000058132	SNORD29	0.008019646	0.836404294
ENSRNOG00000059897	Gjb5	0.008031623	0.897835575
ENSRNOG00000038004	Zfp804a	0.008292877	0.854700333
ENSRNOG00000060952	AABR07056183.1	0.008400321	0.966033997
ENSRNOG00000016671	Dtna	0.008593437	-0.874511241
ENSRNOG00000008481	Reep1	0.008648177	-0.904199386
ENSRNOG00000062071	AABR07005572.2	0.008682534	0.818959385

ENSRNOG00000049676	St6galnac5	0.00876292	-0.943160503
ENSRNOG00000013552	Scd	0.008832035	1.044921621
ENSRNOG00000049959	Igsf21	0.008931909	1.170873362
ENSRNOG00000024954	Mgat5b	0.008951745	-1.111596786
ENSRNOG00000055658	Snord69	0.009035271	1.255846895
ENSRNOG00000010365	Syn1	0.009357656	-0.737938046
ENSRNOG00000056487	Oxr1	0.009569214	-0.856387372
ENSRNOG00000025522	Atf7ip2	0.009779203	0.997291595
ENSRNOG00000031665	Ace2	0.009885766	-1.144692626
ENSRNOG00000059487	AABR07045405.1	0.009918327	-1.125990848
ENSRNOG00000061908	7SK	0.009963107	1.645849516
ENSRNOG00000029859	AABR07021384.1	0.010216625	0.814626597
ENSRNOG00000000105	Cplx2	0.010377176	-2.2140417
ENSRNOG00000014761	Rasd2	0.010411049	-1.002753917
ENSRNOG00000001249	Col6a1	0.010439756	2.618054254
ENSRNOG00000014613	Ddah1	0.010445644	-0.932914561
ENSRNOG00000045749	AC105515.1	0.010759719	1.386754864
ENSRNOG00000030107	AABR07049329.1	0.010914278	0.828287333
ENSRNOG00000047459	H1f4	0.010969004	1.234778657
ENSRNOG00000020532	Kcnq1	0.011041436	0.93499673
ENSRNOG00000008015	Fos	0.011045495	1.192832731
ENSRNOG00000019791	Sipa1l2	0.011213443	-0.745172709
ENSRNOG00000030748	AABR07008421.1	0.01128491	1.031657534
ENSRNOG00000045781	Mir3561	0.011287001	1.221034554
ENSRNOG00000005375	RGD1564053	0.011352957	-0.864856618
ENSRNOG00000021062	Fxyd5	0.011462294	0.938067969
ENSRNOG00000031634	AABR07033920.1	0.011516347	1.08784736
ENSRNOG00000008173	Sesn3	0.011548926	-1.273867467
ENSRNOG00000059344	Tpcn1	0.012039701	-0.853378451
ENSRNOG00000007189	Ttc22	0.012207249	0.99468525
ENSRNOG00000027787	Cdc6	0.012283112	-0.748676703
ENSRNOG00000053850	Rdh5	0.01271603	1.115343014
ENSRNOG00000017597	Fbp1	0.012723744	1.130624415
ENSRNOG00000009884	Lgals1	0.012764759	0.77225133
ENSRNOG00000060434	AABR07053500.2	0.012831646	-1.495947209
ENSRNOG00000061441	AABR07071935.1	0.012854922	0.950338836
ENSRNOG00000001159	Tff3	0.013129001	1.253582665
ENSRNOG00000030214	AABR07029002.1	0.013462432	0.845446572
ENSRNOG00000061205	AABR07032532.2	0.013486062	1.120862413
ENSRNOG00000025394	Tanc1	0.013525568	-0.7714834
ENSRNOG00000016348	Tat	0.01363507	1.054053959
ENSRNOG00000014971	Mas1	0.013680163	-0.950867052
ENSRNOG00000039571	Glod5	0.013719922	0.852626102
ENSRNOG00000020652	Tgfb1	0.014061801	0.82212358

ENSRNOG00000019118	Slc13a3	0.014493827	1.208667798
ENSRNOG00000051196	AABR07018038.1	0.014928641	-0.738120385
ENSRNOG00000057505	AABR07015015.2	0.015150813	1.029370869
ENSRNOG00000029267	LOC103690821	0.015326444	0.922276013
ENSRNOG00000022975	Nfam1	0.01541432	1.066477314
ENSRNOG00000050869	Cebpd	0.015543467	0.894734841
ENSRNOG00000034150	AABR07041778.1	0.015551651	1.037135018
ENSRNOG00000009373	RGD1562690	0.015677456	1.012015384
ENSRNOG00000046144	H3c1	0.016007172	0.922242724
ENSRNOG00000049407	Shisa8	0.016018683	2.163091878
ENSRNOG00000033695	AC095825.1	0.016195458	0.884877459
ENSRNOG00000060502	SNORD96	0.016384663	0.770696898
ENSRNOG00000019931	Map3k21	0.016401138	-1.887915979
ENSRNOG00000009072	AABR07056013.1	0.016674832	0.850416945
ENSRNOG00000012772	Nqo1	0.017015645	-0.81406233
ENSRNOG00000014183	Gnaq	0.017645705	-0.97137295
ENSRNOG00000045646	LOC102549173	0.017811119	0.925359543
ENSRNOG00000012660	Postn	0.017955509	0.774044903
ENSRNOG00000061527	Gck	0.018205201	0.907463059
ENSRNOG00000019902	Folr1	0.018227993	0.886017936
ENSRNOG00000053769	Soat2	0.018253064	0.776764407
ENSRNOG00000021435	AC141966.1	0.018254338	1.02083429
ENSRNOG00000016874	Zfp521	0.018284022	-0.858332122
ENSRNOG00000013547	Slc6a12	0.018482311	0.850472815
ENSRNOG00000018764	B3gnt3	0.018579572	0.895210683
ENSRNOG00000022738	AC098459.1	0.018620224	1.352435683
ENSRNOG00000057557	Prlr	0.018755885	0.93473251
ENSRNOG00000053813	Nkap	0.019213867	-0.858808539
ENSRNOG00000060020	C1ql4	0.01924857	0.864495755
ENSRNOG00000039568	Mgat4e	0.019454509	0.977203243
ENSRNOG00000028895	Rtp4	0.020232604	-1.246833258
ENSRNOG00000001757	Tm4sf19	0.020245164	-0.854572415
ENSRNOG00000003283	Rcsd1	0.0203076	-1.233554885
ENSRNOG00000008398	AABR07055789.1	0.020677697	-1.406820263
ENSRNOG00000032765	AABR07068536.1	0.02073075	1.060755572
ENSRNOG00000000943	AC136867.1	0.020884191	0.828203635
ENSRNOG00000031706	RGD1563601	0.020972114	0.795349148
ENSRNOG00000010362	Anxa2	0.021137396	-0.815586479
ENSRNOG00000027229	Slc35d2	0.021192325	-1.097536749
ENSRNOG00000004874	Flrt3	0.021249715	-1.013011749
ENSRNOG00000048733	Nup62	0.021346236	-0.745254296
ENSRNOG00000024923	Nnat	0.021811206	-1.291039262
ENSRNOG00000031790	H2bu1	0.022048202	1.651093009
ENSRNOG00000055293	Ptprb	0.022288243	0.999377835

ENSRNOG00000004753	Napb	0.022548435	-1.59834726
ENSRNOG00000036856	AC097183.1	0.022604336	0.969685023
ENSRNOG00000050729	LOC102555217	0.023119182	0.847722047
ENSRNOG00000014647	Cbfb	0.023937328	-0.835555525
ENSRNOG00000061479	LOC102555038	0.024001409	-0.99328543
ENSRNOG00000023896	Dusp6	0.024517792	-0.741328638
ENSRNOG00000015854	AABR07051831.1	0.024536008	1.014160318
ENSRNOG00000058899	Snord49a	0.025197233	0.774005158
ENSRNOG00000061979	AC120096.2	0.025260264	0.925159911
ENSRNOG00000020478	Camk4	0.025331601	-0.759794176
ENSRNOG00000014658	Zfp423	0.025476849	0.824814653
ENSRNOG00000047943	LOC108348106	0.02597707	0.810399881
ENSRNOG00000028330	AABR07067600.1	0.025993016	0.795337848
ENSRNOG00000018358	Nt5dc2	0.026205678	0.919165001
ENSRNOG00000030321	LOC502176	0.026589424	0.859189118
ENSRNOG00000011659	Alpk3	0.026634938	0.970724026
ENSRNOG00000046740	LOC688672	0.026905792	0.83983645
ENSRNOG00000029558	Pabpn1l	0.026957694	1.164253148
ENSRNOG00000050706	Col25a1	0.027209733	-1.515651752
ENSRNOG00000004805	Stac2	0.027374469	0.778776999
ENSRNOG00000061925	AABR07059159.1	0.02744146	-1.165921966
ENSRNOG00000049649	Hist1h2ah	0.027529166	0.970218983
ENSRNOG00000022609	Mrps10	0.027914206	0.768179354
ENSRNOG00000049380	AABR07033657.1	0.028288557	0.843022843
ENSRNOG00000008843	Eci1	0.028437962	0.845223485
ENSRNOG00000031590	AABR07048308.2	0.029714311	1.057491706
ENSRNOG00000021581	Rapgef2	0.029789128	-0.79785118
ENSRNOG00000008658	Mitf	0.029967239	-0.806062659
ENSRNOG00000003266	LOC100361655	0.030381013	0.837965535
ENSRNOG00000060395	AABR07025301.1	0.03099389	1.0962684
ENSRNOG00000010484	Zdhhc21	0.031038805	-0.948472993
ENSRNOG00000028845	Ebf1	0.031441661	-0.963123636
ENSRNOG00000013018	Eda2r	0.031584715	-1.89489354
ENSRNOG00000005576	Rpia	0.031784003	-0.807314443
ENSRNOG00000053620	AABR07051069.1	0.031951499	1.23927566
ENSRNOG00000041826	AABR07053152.1	0.032029924	0.931789451
ENSRNOG00000018237	Gstp1	0.032809234	-0.75060468
ENSRNOG00000014197	Tmem51	0.033947845	0.90161508
ENSRNOG00000056485	LOC103694903	0.03526823	0.889740777
ENSRNOG00000001227	Adarb1	0.035435376	-0.82285646
ENSRNOG00000022595	LOC100362965	0.035665407	0.909335358
ENSRNOG00000048834	Plin3	0.035673383	2.469908258
ENSRNOG00000016336	AABR07045373.1	0.035824981	0.901242372
ENSRNOG00000056942	SNORD31	0.036018411	0.896850727

ENSRNOG00000018109	Clic4	0.036122429	-1.626583958
ENSRNOG00000062002	Kcna3	0.03645952	-1.504050996
ENSRNOG00000011854	Dennd11	0.036500705	-1.283600543
ENSRNOG00000032947	AABR07067749.1	0.036706906	1.022584921
ENSRNOG00000001123	RGD1562310	0.037433464	-0.963573847
ENSRNOG00000023068	Cd5l	0.037522246	-1.022953597
ENSRNOG00000020129	Cdh3	0.038206721	-0.842827704
ENSRNOG00000042753	Fgf13	0.038232128	-0.886021562
ENSRNOG00000031669	Lpp	0.038481229	1.251049741
ENSRNOG00000042752	AABR07065010.1	0.038527852	0.884240728
ENSRNOG00000060156	SNORD28	0.03963364	0.771664341
ENSRNOG00000019316	Sh3bp4	0.040852495	-0.873162313
ENSRNOG00000048585	AABR07018244.1	0.041173647	0.785360918
ENSRNOG00000011642	Tfap2d	0.041473238	-0.774298301
ENSRNOG00000011009	Cmtm4	0.041489733	-1.523468078
ENSRNOG00000062228	LOC100359515	0.04229718	-1.776535531
ENSRNOG00000062246	AABR07003509.1	0.042651163	1.004497662
ENSRNOG00000021745	Bhlhe22	0.043069871	-1.19541723
ENSRNOG00000024259	Tmem54	0.043628354	0.801407158
ENSRNOG00000015848	Etfrf1	0.043683776	0.944974267
ENSRNOG00000049985	Gprasp1	0.044773032	-0.810227945
ENSRNOG00000046181	Anks4b	0.044787123	-0.746744011
ENSRNOG00000058136	Vom1r90	0.04494678	-1.496734821
ENSRNOG00000030299	AABR07024742.1	0.046101202	0.983916875
ENSRNOG00000030447	AC105662.1	0.046183363	0.898049883
ENSRNOG00000048812	Gpx1	0.046302472	0.981769305
ENSRNOG00000033673	AABR07061532.1	0.047188744	1.21691595
ENSRNOG00000017842	Scnn1g	0.047287865	0.820718925
ENSRNOG00000032148	AABR07018050.1	0.047753835	1.18005106
ENSRNOG00000006950	Padi3	0.047903076	0.861607138
ENSRNOG00000055292	LOC679087	0.048127967	0.851137352
ENSRNOG00000036665	AC110474.1	0.048434055	1.088681656
ENSRNOG00000006033	LOC100910790	0.048502184	-0.759955971
ENSRNOG00000042905	RT1-T24-4	0.049861467	0.862123419

Supplementary Table S3 (set 3 differentially expressed genes)

Ensemble ID	Gene name	p-value	log2FoldChange
ENSRNOG00000002413	Gpc4	4.58501E-17	2.188752181
ENSRNOG00000026605	Ifi27l2b	6.57965E-16	-2.162404371
ENSRNOG00000000825	Calhm5	1.59635E-13	4.220348562
ENSRNOG00000009031	Gucy2c	1.65021E-12	3.014197153
ENSRNOG00000012906	Bcas1	2.66001E-12	-4.438778076

ENSRNOG00000037188	Mug1	1.00636E-09	4.3341293
ENSRNOG00000020325	Calhm2	1.2174E-09	2.145719468
ENSRNOG00000032699	Ttyh1	3.77758E-09	1.780735551
ENSRNOG00000027767	Slc38a5	2.97848E-08	3.272845012
ENSRNOG00000045919	LOC103694120	3.19256E-08	5.662724359
ENSRNOG00000007539	Rsad2	3.47387E-08	-1.858110862
ENSRNOG00000013166	Wnt4	5.5766E-08	2.06127471
ENSRNOG00000024517	Gpr119	9.86361E-08	2.53937917
ENSRNOG00000025055	Zdhhc19	5.08162E-07	-4.17764389
ENSRNOG00000010011	Osbpl3	5.49993E-07	-1.660623302
ENSRNOG00000021802	Isg15	6.14088E-07	-1.6308009
ENSRNOG00000000463	Col11a2	6.90978E-07	-1.338591538
ENSRNOG00000022921	Dact2	7.67474E-07	1.708545282
ENSRNOG00000017427	LOC100909840	9.8389E-07	4.305869045
ENSRNOG00000001187	Oasl	1.60515E-06	-1.41749886
ENSRNOG00000031167	AABR07054319.1	1.78335E-06	5.135476384
ENSRNOG00000053055	Otop3	3.11692E-06	4.04106576
ENSRNOG00000037167	Rtp3	3.51193E-06	-1.548953382
ENSRNOG00000021507	Luzp2	4.39524E-06	2.25268066
ENSRNOG00000006280	Pcsk9	4.91755E-06	2.423760595
ENSRNOG00000059232	U6	4.95313E-06	-4.759441514
ENSRNOG00000022256	Cxcl10	5.20296E-06	-1.964640147
ENSRNOG00000013092	Lonrf3	5.83416E-06	1.158044512
ENSRNOG00000033722	Rnf207	5.93489E-06	-2.108479092
ENSRNOG00000021011	Fut2	1.05141E-05	1.682484759
ENSRNOG00000012417	Iapp	1.13441E-05	2.348244856
ENSRNOG00000022267	AABR07039334.1	1.26177E-05	4.037531898
ENSRNOG00000030954	Fat1	1.37827E-05	1.241846984
ENSRNOG00000046404	LOC291276	1.49036E-05	4.198409199
ENSRNOG00000010725	Cpa1	1.49561E-05	-1.900350831
ENSRNOG00000017525	Epha1	2.0835E-05	2.299636095
ENSRNOG00000015075	Stc1	2.18714E-05	2.288001872
ENSRNOG00000007048	Rap1b	2.21757E-05	1.354085254
ENSRNOG00000053334	Stmn4	2.63359E-05	-1.449545344
ENSRNOG00000008337	Gjd2	3.28199E-05	2.078994973
ENSRNOG00000011411	Adgrg6	3.47326E-05	4.581623077
ENSRNOG00000020792	Etv4	3.53519E-05	-1.728493476
ENSRNOG00000006331	Elovl5	3.8725E-05	2.570325019
ENSRNOG00000000055	Fcrl6	6.00224E-05	-3.05982555
ENSRNOG00000012052	Ins1	6.11068E-05	1.592583804
ENSRNOG00000010758	Lpar2	6.66505E-05	4.797522219
ENSRNOG00000001849	Mapk1	6.72366E-05	1.385324929
ENSRNOG00000022537	E2f8	6.73713E-05	2.002873506
ENSRNOG00000001640	Tomm70	6.85445E-05	1.469914054

ENSRNOG00000028895	Rtp4	6.99626E-05	-1.940757465
ENSRNOG00000004192	Arhgap30	7.55298E-05	2.337079711
ENSRNOG00000018420	Slc22a7	7.58136E-05	2.125371002
ENSRNOG00000004089	Enpp2	7.75426E-05	2.130171251
ENSRNOG00000016983	Myh7	7.75986E-05	6.169814497
ENSRNOG00000015550	Ptgds	8.83909E-05	-1.204667149
ENSRNOG00000013304	Arg1	8.97845E-05	-1.545457031
ENSRNOG00000053706	Lonrf1	9.28315E-05	2.945033282
ENSRNOG00000034230	Fcrl1	9.45911E-05	-3.254763907
ENSRNOG00000053044	AABR07012775.1	9.81482E-05	-2.598016137
ENSRNOG00000054282	AABR07072203.1	0.000103454	-4.104204783
ENSRNOG00000008536	Actc1	0.000118065	-2.669871756
ENSRNOG00000008979	Guca2b	0.000128776	-1.962152489
ENSRNOG00000000021	AABR07061902.1	0.000136196	4.025341129
ENSRNOG00000015376	Npas1	0.000141301	3.391721132
ENSRNOG00000049437	Gpc1	0.000141863	2.409340816
ENSRNOG00000001729	Xxylt1	0.000150979	4.151378454
ENSRNOG00000013443	Tm9sf3	0.000153106	1.37948961
ENSRNOG00000050869	Cebpd	0.000155215	1.361173306
ENSRNOG00000008000	Syt13	0.000155687	0.950581949
ENSRNOG00000000595	Traf3ip2	0.000158242	-1.136827308
ENSRNOG00000009373	RGD1562690	0.00017358	2.076321508
ENSRNOG00000003025	AABR07039336.2	0.000177387	1.579284286
ENSRNOG00000005486	Phf20l1	0.000180502	-1.982287168
ENSRNOG00000002802	Cxcl1	0.000181711	-2.143052942
ENSRNOG00000004100	Trib1	0.000193138	3.097708506
ENSRNOG00000007514	Sox12	0.000219318	3.56098831
ENSRNOG00000047712	Dcx	0.000223468	-2.321898915
ENSRNOG00000046327	Rbpj	0.000228377	1.045255301
ENSRNOG00000054172	Ctnnb1	0.000231711	2.361192115
ENSRNOG00000005369	Kcnj3	0.000232178	1.816501479
ENSRNOG00000047628	Khsrp	0.000246502	2.458963269
ENSRNOG00000058710	AABR07021465.2	0.000248472	2.803310989
ENSRNOG00000046727	Abcc2	0.000249981	-1.416213681
ENSRNOG00000012881	Fgl2	0.000252991	1.3522542
ENSRNOG00000022800	Sp140	0.000256991	-1.508302625
ENSRNOG00000025742	Lmnb2	0.000258279	3.320084874
ENSRNOG00000009323	Fam214b	0.000268898	1.664833686
ENSRNOG00000019598	Vegfa	0.000269698	1.182043369
ENSRNOG00000021176	Mtmr11	0.000270887	-0.87507157
ENSRNOG00000010362	Anxa2	0.000282296	-1.078915621
ENSRNOG00000048961	Bhlhe41	0.000295509	3.58361496
ENSRNOG00000014288	Fn1	0.000296053	1.763506511
ENSRNOG00000026120	Fam8a1	0.000311159	2.918646847

ENSRNOG00000056148	Nudcd3	0.00031237	3.151835563
ENSRNOG00000001212	Dnmt3l	0.000315043	-2.607145129
ENSRNOG00000062101	Ace	0.000334486	3.408858348
ENSRNOG00000009514	Mme	0.00033502	1.102553329
ENSRNOG00000014689	Zbtb4	0.000344844	3.947537491
ENSRNOG00000011124	AABR07067267.1	0.000367178	1.552796995
ENSRNOG00000012728	Nkx2-2	0.000373996	1.653786995
ENSRNOG00000015406	Pgm5	0.000392929	3.070888867
ENSRNOG00000009884	Lgals1	0.000395637	1.255198528
ENSRNOG00000004692	A1bg	0.000396079	1.695638808
ENSRNOG00000017434	Mgat3	0.000400198	3.272787024
ENSRNOG00000000781	Rnf39	0.000401109	1.322378303
ENSRNOG00000056716	Zbtb20	0.000410906	-1.891844258
ENSRNOG00000012393	S100a13	0.000415094	-1.328717303
ENSRNOG00000061998	AABR07044421.1	0.000444398	3.392731965
ENSRNOG00000032414	Tmem161b	0.000451439	1.20955013
ENSRNOG00000059142	Dtwd2	0.000454257	2.132384377
ENSRNOG00000013798	Fnbp1l	0.000456282	1.103038764
ENSRNOG00000026953	Gpr88	0.000467039	1.023904524
ENSRNOG00000007668	Mafa	0.000477601	5.515978927
ENSRNOG00000014035	Arhgef4	0.000481903	4.020473126
ENSRNOG00000020295	Plekhn1	0.000484291	-1.272752271
ENSRNOG00000012054	Zmpste24	0.000487498	1.196737945
ENSRNOG00000036701	Actg1	0.000489285	2.430125298
ENSRNOG00000002934	Atp1b1	0.000492925	1.047678097
ENSRNOG00000049407	Shisa8	0.00049517	3.359676507
ENSRNOG00000060229	Arf1	0.000497172	1.886037884
ENSRNOG00000009019	Slc6a6	0.000503533	2.007122893
ENSRNOG00000013452	Rcn1	0.000508687	2.050508462
ENSRNOG00000048875	Znrf4	0.000509135	3.135678353
ENSRNOG00000003769	Tmem163	0.000510142	1.231611944
ENSRNOG00000058545	Arhgap4	0.000531485	-1.078211572
ENSRNOG00000038572	Ncapg	0.00053304	1.176664948
ENSRNOG00000019295	Rab12	0.000546586	2.758796256
ENSRNOG00000060063	Naa10	0.000546843	-1.138640368
ENSRNOG00000016704	Pcyox1	0.000559853	2.304317216
ENSRNOG00000053813	Nkap	0.00056608	-1.860679233
ENSRNOG00000021128	Kcnj11	0.000571218	1.030698114
ENSRNOG00000045770	Hira	0.000572234	4.277212635
ENSRNOG00000029360	Serinc1	0.000573829	1.782291276
ENSRNOG00000024832	Gpr158	0.000581247	2.62771344
ENSRNOG00000020557	Ryr1	0.000588945	-1.164631974
ENSRNOG00000024492	Ap1ar	0.000606003	2.176091497
ENSRNOG00000031113	Zscan22	0.000607637	3.038969337

ENSRNOG00000006987	Tmem263	0.000608429	2.055539454
ENSRNOG00000019730	Inppl1	0.000619486	3.024861867
ENSRNOG00000045738	Ak4	0.000648723	3.654464482
ENSRNOG00000004500	Myc	0.000649907	1.417332524
ENSRNOG00000053452	LOC100361457	0.000653115	1.171597555
ENSRNOG00000033280	Pam	0.000655454	1.005570647
ENSRNOG00000047453	Casd1	0.000656812	1.950152641
ENSRNOG00000008553	Mthfr	0.000659947	4.082065992
ENSRNOG00000010813	Tspan14	0.000667321	2.398698937
ENSRNOG00000009856	Sypl1	0.000685389	1.074712159
ENSRNOG00000022941	AABR07004746.1	0.000693127	2.063046149
ENSRNOG00000042499	LOC100364435	0.000696681	-1.216183187
ENSRNOG00000003832	Vash2	0.000709825	-1.349712346
ENSRNOG00000015297	RGD1561662	0.000712086	2.095423101
ENSRNOG00000039587	Wdr13	0.00071874	2.505960852
ENSRNOG00000017429	Lat	0.000719881	-1.112753376
ENSRNOG00000005308	Tmx2	0.000727603	1.302348268
ENSRNOG00000061316	Ulbp1	0.000730756	1.544092451
ENSRNOG00000015354	Aox1	0.000756421	-1.089818885
ENSRNOG00000013851	Spry4	0.000764145	-1.204956678
ENSRNOG00000010880	Gpr27	0.000770023	2.76617284
ENSRNOG00000022738	AC098459.1	0.000793602	2.717785304
ENSRNOG00000020930	Atxn7l3	0.000821801	2.736205435
ENSRNOG00000013946	Rnf149	0.000850013	2.642713188
ENSRNOG00000022698	Vsig10	0.000853243	2.583753225
ENSRNOG00000007561	Glb1l2	0.000871088	2.377141453
ENSRNOG00000004909	Cnot2	0.000892985	1.041806992
ENSRNOG00000020261	Fam53c	0.000926528	3.215234987
ENSRNOG00000025074	Fgg	0.000944444	1.836162923
ENSRNOG00000009282	Nub1	0.000951727	2.175437103
ENSRNOG00000005130	LOC103693780	0.000953745	2.222141145
ENSRNOG00000019613	Syt9	0.000964025	2.458906789
ENSRNOG00000005498	Gcg	0.000971271	2.347458579
ENSRNOG00000011287	Minpp1	0.000977482	2.054594595
ENSRNOG00000001217	Lrrc3	0.000983738	3.415324558
ENSRNOG00000006832	Zdhhc5	0.000986825	1.848010225
ENSRNOG00000055246	Ncor1	0.000991187	-1.488146176
ENSRNOG00000023467	Fam168b	0.000993149	1.786942787
ENSRNOG00000004405	Pigr	0.001000622	2.568677011
ENSRNOG00000016950	Otud1	0.001000715	3.285101664
ENSRNOG00000015177	Sun2	0.001007885	3.067686628
ENSRNOG00000013547	Slc6a12	0.001024118	2.185872921
ENSRNOG00000005260	Acp1	0.001026019	1.337043874
ENSRNOG00000005818	RGD1563349	0.001032079	1.246595502

ENSRNOG00000027592	Rerg	0.001043407	-2.021317058
ENSRNOG00000006241	Marchf7	0.001047644	1.935154854
ENSRNOG00000057096	Gnai1	0.001049009	2.305116002
ENSRNOG00000019992	AABR07054400.1	0.001050816	2.102703691
ENSRNOG00000010109	Nol9	0.001060044	2.439650563
ENSRNOG00000010064	Abcc4	0.001113202	2.19193811
ENSRNOG00000061544	Spock2	0.001125128	3.048550645
ENSRNOG00000003134	Slc4a4	0.001129442	2.849514593
ENSRNOG00000051420	Cables2	0.001138848	2.561473611
ENSRNOG00000007759	Foxd2	0.001147955	2.877590509
ENSRNOG00000005191	Trim44	0.00115455	2.27560385
ENSRNOG00000049349	Tafa5	0.001155657	-1.298442464
ENSRNOG00000004628	Dazap2	0.001160376	1.214079927
ENSRNOG00000049496	Trim62	0.001160475	3.456556166
ENSRNOG00000017974	Gprin1	0.001173997	3.314194002
ENSRNOG00000012106	Dnaja4	0.001175042	1.190152399
ENSRNOG00000006600	Unk	0.001185754	2.219992606
ENSRNOG00000007034	Hipk2	0.001188312	-1.45077923
ENSRNOG00000042860	Pappa2	0.001193301	1.164116872
ENSRNOG00000019346	LOC103693999	0.001194051	1.417038025
ENSRNOG00000010853	Chrna7	0.001202412	2.713046764
ENSRNOG00000003189	Cited1	0.001208938	-0.98344466
ENSRNOG00000042340	LOC679894	0.001209239	-2.212360678
ENSRNOG00000009625	Dpysl2	0.001233555	2.471015741
ENSRNOG00000011417	Pde3b	0.001251181	1.311602918
ENSRNOG00000050445	Gde1	0.001256867	2.161372679
ENSRNOG00000018803	Mrtfa	0.001261095	2.681017529
ENSRNOG00000015428	Mff	0.001267765	2.356186635
ENSRNOG00000024027	Lemd3	0.001268099	1.169428917
ENSRNOG00000055446	Amfr	0.001278577	2.757032685
ENSRNOG00000021438	Tuba1c	0.001291497	2.717717059
ENSRNOG00000004423	Zbtb18	0.001292395	3.109246328
ENSRNOG00000025669	Tmem104	0.001294831	3.461843235
ENSRNOG00000028733	Prkar1b	0.001297267	2.696197159
ENSRNOG00000025349	Maneal	0.001311656	2.790524059
ENSRNOG00000019958	Tmem151b	0.001319545	3.243294469
ENSRNOG00000014675	Pi4k2a	0.001344345	2.590495309
ENSRNOG00000014209	Utp6	0.001345434	2.228921531
ENSRNOG00000000606	Pcdh15	0.001349611	2.531442931
ENSRNOG00000010298	Xbp1	0.001355903	1.262936609
ENSRNOG00000015970	Tbc1d13	0.001361226	2.408438006
ENSRNOG00000002433	G3bp2	0.001387969	1.887301363
ENSRNOG00000014504	Il1r1	0.001389842	2.005706855
ENSRNOG00000059373	LOC100909548	0.001426395	2.167839948

ENSRNOG00000015502	Mfsd14a	0.001446968	1.833281944
ENSRNOG00000012742	Irx2	0.001460985	2.145506096
ENSRNOG00000002781	Kdsr	0.001467008	1.525194557
ENSRNOG00000008478	Mmp13	0.001467362	2.65819623
ENSRNOG00000019560	Pde2a	0.001487085	1.58867519
ENSRNOG00000050337	AABR07054264.1	0.001496738	-3.370641527
ENSRNOG00000014496	Coro6	0.001502287	-0.970066565
ENSRNOG00000048818	Catsperd	0.001513277	-1.529763245
ENSRNOG00000013763	Erlin2	0.001516105	1.148919446
ENSRNOG00000016863	Pnma8b	0.001545098	2.394683534
ENSRNOG00000010960	Ankh	0.001546015	1.71185457
ENSRNOG00000013474	Casz1	0.001549428	2.508878588
ENSRNOG00000011363	Napepld	0.001553943	2.83684265
ENSRNOG00000004339	Yy1	0.001559301	1.686160777
ENSRNOG00000024712	Insc	0.001576647	-2.43783293
ENSRNOG00000018987	Crebzf	0.001579641	2.674745539
ENSRNOG00000020259	RGD1305347	0.001613718	1.250318751
ENSRNOG00000031930	Bin2	0.001621099	-2.169096154
ENSRNOG00000018671	Commd4	0.001621851	-0.809952155
ENSRNOG00000005758	Btbd11	0.001635835	2.990495692
ENSRNOG00000009470	Flnb	0.001652186	2.845758726
ENSRNOG00000011552	Mon1b	0.00165349	2.335162702
ENSRNOG00000020440	Fads2	0.001654378	2.009456732
ENSRNOG00000051720	Nudc	0.00165645	3.086408099
ENSRNOG00000049484	Atp9a	0.001666824	2.683182902
ENSRNOG00000011007	Ube2o	0.001704621	2.969987726
ENSRNOG00000006868	Ube2z	0.001710314	2.576460983
ENSRNOG00000014168	Xylb	0.001736981	-1.066051015
ENSRNOG00000055962	Bgn	0.001788355	-1.257811828
ENSRNOG00000003736	Col5a2	0.001792125	-2.299760219
ENSRNOG00000054257	Adam10	0.001824286	1.654721213
ENSRNOG00000023492	Greb1l	0.001837784	2.610389279
ENSRNOG00000000488	Hmga1	0.001838747	3.100400085
ENSRNOG00000004332	Egfr	0.001847304	2.045734287
ENSRNOG00000010172	Mkrn3	0.001848023	2.106860242
ENSRNOG00000055307	AABR07058886.1	0.001858216	2.691055209
ENSRNOG00000000175	Mier2	0.001864857	2.118243339
ENSRNOG00000017428	Map1b	0.00188205	-1.4149296
ENSRNOG00000012653	Ppp1r13b	0.001900151	2.078823751
ENSRNOG00000021292	AABR07028027.1	0.001904208	1.10751477
ENSRNOG00000005616	Ncoa3	0.001909942	2.425700746
ENSRNOG00000017231	Adam9	0.001953085	1.463806574
ENSRNOG00000008196	Parp12	0.0019552	2.452437227
ENSRNOG00000010076	Pkp1	0.002006747	1.739602741

ENSRNOG00000047436	Cabyr	0.002047699	-1.685128544
ENSRNOG00000055293	Ptprb	0.002048096	1.764519803
ENSRNOG00000020084	Pcdhb5	0.002060404	1.885148334
ENSRNOG00000045884	Gm25994	0.002069235	1.745075997
ENSRNOG00000014605	Lig4	0.002084637	1.689064988
ENSRNOG00000008051	Itpk1	0.002130503	2.076874237
ENSRNOG00000009263	Ifi27	0.002139241	-0.959256804
ENSRNOG00000007722	Tlcd3a	0.00214115	1.114966285
ENSRNOG00000002414	Tfcp2l1	0.002161012	1.936330176
ENSRNOG00000005331	Vapb	0.002174361	1.705478034
ENSRNOG00000009173	Smad6	0.002206297	2.402691658
ENSRNOG00000004425	Klhdc1	0.002215965	-1.683259244
ENSRNOG00000028460	Lrrc8e	0.002270575	3.042114402
ENSRNOG00000018229	Slc45a1	0.00227084	-2.018833176
ENSRNOG00000058733	Asap1	0.002277152	1.174502229
ENSRNOG00000042560	Bag4	0.002277258	2.061856726
ENSRNOG00000006144	Cntn3	0.002278107	1.34520577
ENSRNOG00000031090	RT1-CE7	0.002290385	2.7487834
ENSRNOG00000036742	Uqcrc2	0.002308173	1.168672819
ENSRNOG00000050108	LOC100911319	0.002308594	3.378744862
ENSRNOG00000026791	Pgbd5	0.002311387	2.765475164
ENSRNOG00000060010	Ss18l1	0.002311816	3.031954543
ENSRNOG00000028422	Rmnd5a	0.00237342	1.664936846
ENSRNOG00000019525	Hspa9	0.002375524	1.632663268
ENSRNOG00000030639	Usp13	0.002419907	-1.090275528
ENSRNOG00000007518	Nckap1	0.002455109	0.911524736
ENSRNOG00000047306	Stap2	0.002491367	2.667542331
ENSRNOG00000005763	Arl1	0.002511326	0.99211442
ENSRNOG00000015676	Brd9	0.00254191	-0.793447063
ENSRNOG00000021380	Fads6	0.002564356	2.957258831
ENSRNOG00000016207	Galnt1	0.002574215	1.369582745
ENSRNOG00000009609	LOC299312	0.002592325	1.785032907
ENSRNOG00000020995	Fut1	0.002618742	1.762364165
ENSRNOG00000016483	Myo16	0.00262008	0.968817429
ENSRNOG00000014354	Cep95	0.002620697	-0.919694293
ENSRNOG00000017307	Prss23	0.002633522	0.887288032
ENSRNOG00000028576	AABR07028446.1	0.002653132	-1.596839241
ENSRNOG00000025811	Cfp	0.002653137	-0.986126348
ENSRNOG00000007490	Gabrr2	0.002656796	-4.112375579
ENSRNOG00000043311	Birc7	0.002668141	-1.165122944
ENSRNOG00000008526	Pdzd3	0.002721484	-1.247032973
ENSRNOG00000002807	Septin9	0.002733859	2.490129997
ENSRNOG00000018677	Akt2	0.002740344	2.083371612
ENSRNOG00000015496	Tpm4	0.002778125	1.741282633

ENSRNOG00000005176	Map7d2	0.00278733	-1.507916341
ENSRNOG00000031662	Slc6a5	0.002828189	1.144262492
ENSRNOG00000027719	AABR07068154.1	0.002830669	3.960965105
ENSRNOG00000057378	Clec16a	0.002841423	1.92374998
ENSRNOG00000060723	AABR07071244.1	0.00285722	1.647331029
ENSRNOG00000008105	Dmrta2	0.002863677	2.788313865
ENSRNOG00000006761	Sh3gl2	0.002870878	2.382227674
ENSRNOG00000025509	Apbb2	0.00287209	2.126438531
ENSRNOG00000046497	Brap	0.002877073	2.163929903
ENSRNOG00000006131	Mettl2	0.002882494	1.640231807
ENSRNOG00000018788	Btbd2	0.002911526	2.59785921
ENSRNOG00000010448	Ptbp1	0.002955592	1.200995828
ENSRNOG00000012394	Bcl2l13	0.003018039	2.671068889
ENSRNOG00000016437	Tm4sf4	0.003018562	1.014903637
ENSRNOG00000029592	Srsf11	0.003058217	-1.469859127
ENSRNOG00000028713	Acvrl1	0.003073815	1.787446564
ENSRNOG00000014076	Mbnl1	0.003080779	1.812190941
ENSRNOG00000009284	Foxa1	0.003104287	2.549899873
ENSRNOG00000003234	Mgrn1	0.003118922	2.176594588
ENSRNOG00000004747	Slc30a8	0.003129085	1.25930873
ENSRNOG00000048580	Trip6	0.003132649	-0.897023947
ENSRNOG00000013734	Dnai1	0.003162448	-1.530880079
ENSRNOG00000056768	AABR07035428.2	0.003174596	-0.979214111
ENSRNOG00000007727	Lhfpl4	0.003189301	2.711005933
ENSRNOG00000036813	#REF!	0.003190468	1.361048161
ENSRNOG00000005258	Myef2	0.003197979	1.186342576
ENSRNOG00000016302	Cnnm1	0.003220381	2.261743579
ENSRNOG00000030445	Ormdl3	0.003235617	2.323670453
ENSRNOG00000022922	Slc25a12	0.003237121	1.34455494
ENSRNOG00000042258	RGD1561157	0.003247457	3.417107235
ENSRNOG00000012236	Hddc3	0.003259592	1.104000207
ENSRNOG00000008400	Pml	0.003262983	2.777346847
ENSRNOG00000048095	RGD1564801	0.003280841	1.650133703
ENSRNOG00000048088	Mest	0.003288654	1.568824181
ENSRNOG00000020857	Ppfia1	0.003293285	2.280219181
ENSRNOG00000019129	Fcgbp	0.003296846	1.450225057
ENSRNOG00000025890	Opa3	0.003299717	1.97861621
ENSRNOG00000029996	AABR07054368.1	0.003314324	1.296789553
ENSRNOG00000006828	Baz1a	0.003344978	-1.419060222
ENSRNOG00000011705	Stmn2	0.003347395	-1.048473004
ENSRNOG00000030597	Ankrd52	0.003370894	2.092428856
ENSRNOG00000018603	Carns1	0.003370911	2.680912481
ENSRNOG00000026435	Arid3a	0.003386952	1.723630375
ENSRNOG00000011748	S100a5	0.003401476	-2.019776645

ENSRNOG00000047257	Tmem185b	0.003430929	1.091552079
ENSRNOG00000005766	Tbc1d20	0.003550681	2.15057556
ENSRNOG00000005366	AABR07053472.1	0.003603938	1.730468265
ENSRNOG00000060005	Surf4	0.003615574	1.292443004
ENSRNOG00000012807	C1qa	0.003647058	2.199323493
ENSRNOG00000056041	AABR07062570.1	0.003677289	1.823314665
ENSRNOG00000007971	Wbp2	0.003679797	1.962816304
ENSRNOG00000013963	#N/A	0.003700283	2.11733869
ENSRNOG00000056940	Cited2	0.00370282	1.004041579
ENSRNOG00000023768	Rundc1	0.003713864	2.096830526
ENSRNOG00000023969	Herc6	0.003775412	-1.39198045
ENSRNOG00000058044	U6	0.003788711	-2.846791771
ENSRNOG00000060410	Pcdh1	0.003796823	1.863839954
ENSRNOG00000013190	Rnaset2	0.003813821	-0.741585515
ENSRNOG00000057284	Cenpb	0.0038324	2.029766882
ENSRNOG00000004783	Fam171b	0.003889275	2.271814676
ENSRNOG00000026748	Dennd2a	0.003904832	-0.868381545
ENSRNOG00000009198	Rab6b	0.003927402	-0.873647295
ENSRNOG00000027286	Lrrc75a	0.003931536	2.852032563
ENSRNOG00000008053	Atp8a2	0.0039396	1.376534833
ENSRNOG00000004072	Myo1c	0.003956506	2.177033501
ENSRNOG00000003779	Dedd	0.003991114	1.1364012
ENSRNOG00000002408	Rbm47	0.004021426	2.351790759
ENSRNOG00000004317	Vipr2	0.004037572	-0.94768655
ENSRNOG00000016976	Clcn7	0.004047691	2.612319576
ENSRNOG00000049402	Nbl1	0.004059245	1.879662336
ENSRNOG00000022609	Mrps10	0.00407473	1.404729536
ENSRNOG00000002191	LOC498368	0.004092721	0.946386851
ENSRNOG00000007990	Adipor2	0.004101633	1.851680323
ENSRNOG00000019244	Mxra8	0.004140552	1.134661042
ENSRNOG00000031643	Dchs1	0.004144718	1.613555718
ENSRNOG00000002871	Rbm25l1	0.004159392	-1.592700675
ENSRNOG00000009577	Ndst4	0.004222111	-1.569741471
ENSRNOG00000055672	Gpx2	0.004225096	-2.46054293
ENSRNOG00000055411	AC128836.1	0.004264927	-1.70628034
ENSRNOG00000017912	Atp2a3	0.004265288	1.019634952
ENSRNOG00000020640	Zbtb7b	0.004269088	2.409659468
ENSRNOG00000042728	Fbrs	0.004288046	2.185645189
ENSRNOG00000020353	Sh3pxd2a	0.004305626	2.90158201
ENSRNOG00000006719	Zfp618	0.004306981	-1.435798463
ENSRNOG00000020073	Pcdhb8	0.004348768	2.271122926
ENSRNOG00000057056	Rab5a	0.004349235	2.070378876
ENSRNOG00000004111	Soat1	0.004358841	1.66186638
ENSRNOG00000022771	Arhgap23	0.004359154	2.986131237

ENSRNOG00000010232	Enpp5	0.004362465	0.853060023
ENSRNOG00000015888	Larp4b	0.004392973	2.159385796
ENSRNOG00000002874	AABR07065078.1	0.00443012	-1.439843766
ENSRNOG00000019807	Sufu	0.004444051	1.919393639
ENSRNOG00000018698	Wac	0.004447815	1.135732208
ENSRNOG00000012017	Otulin	0.004468208	2.398291188
ENSRNOG00000018824	Slc7a5	0.004476647	0.857178375
ENSRNOG00000053859	Ranbp6	0.004494559	1.609096569
ENSRNOG00000057290	Tmem168	0.004501049	2.079248342
ENSRNOG00000058136	Vom1r90	0.004507171	-1.830069309
ENSRNOG00000042286	#N/A	0.004545315	1.799300156
ENSRNOG00000049633	Flvcr1	0.004557884	2.522640494
ENSRNOG00000016472	Marchf4	0.004558675	2.513232551
ENSRNOG00000042101	Zfp93	0.004573168	1.884949979
ENSRNOG00000013847	Nova2	0.004628492	2.056902848
ENSRNOG00000013520	Mat2a	0.004674437	0.891969074
ENSRNOG00000020833	Cspg5	0.004692549	1.889659398
ENSRNOG00000014751	Ret	0.004709687	1.371435719
ENSRNOG00000011599	Gldc	0.004728871	-1.430174976
ENSRNOG00000009478	RGD1359108	0.004762522	2.042601134
ENSRNOG00000032180	Ccdc15	0.004781642	-2.24688177
ENSRNOG00000059202	AABR07021988.1	0.004783181	0.864261007
ENSRNOG00000011026	Irf2bpl	0.004793213	1.807265354
ENSRNOG00000003953	Rab3gap1	0.004793741	2.029573059
ENSRNOG00000011713	LOC691113	0.004824683	2.611785939
ENSRNOG00000059840	Unc5a	0.004855816	2.778750487
ENSRNOG00000015936	LOC108349548	0.004870737	-0.847674785
ENSRNOG00000006860	Itk	0.00488428	-2.475062351
ENSRNOG00000019639	Pgpep1	0.004892362	1.274643222
ENSRNOG00000055082	Maz	0.004905352	2.408656804
ENSRNOG00000011518	Dusp26	0.004930813	-0.782863837
ENSRNOG00000011151	Tenm4	0.004935694	2.299212633
ENSRNOG00000010479	Mycbp2	0.004937434	-1.102269396
ENSRNOG00000018406	Wipf1	0.004940165	1.001967456
ENSRNOG00000017265	#N/A	0.00496414	1.51372684
ENSRNOG00000003870	C1qtnf2	0.004998866	1.791033582
ENSRNOG00000000321	Cd24	0.005033679	1.227851033
ENSRNOG00000004678	Itln1	0.005042261	0.836377751
ENSRNOG00000029614	Robo1	0.005070155	2.469882242
ENSRNOG00000000502	Def6	0.005104742	1.070979244
ENSRNOG00000009957	Slc25a13	0.005203483	2.190755948
ENSRNOG00000032735	Srek1	0.005229859	-0.982784419
ENSRNOG00000058132	SNORD29	0.005274824	1.134362242
ENSRNOG00000022043	Yipf2	0.005282989	1.384351276

ENSRNOG00000019321	Cck	0.005295871	-0.758785447
ENSRNOG00000019869	Lrfn1	0.005312176	2.265877189
ENSRNOG00000032639	Foxo6	0.005347617	2.569069214
ENSRNOG00000005291	Slc38a1	0.005363375	1.053469535
ENSRNOG00000037227	Yes1	0.005373305	2.175449049
ENSRNOG00000013959	Skor1	0.005389297	2.379914364
ENSRNOG00000062228	LOC100359515	0.0054061	-2.262490827
ENSRNOG00000028206	Pheta1	0.005458825	2.855432001
ENSRNOG00000003143	RGD1310166	0.005545438	1.356946107
ENSRNOG00000008065	Wwc1	0.005563791	2.320665151
ENSRNOG00000002176	Nectin3	0.005585745	1.236368604
ENSRNOG00000024578	Ttyh2	0.005599297	2.607840444
ENSRNOG00000037080	Adamts17	0.005601918	1.88564959
ENSRNOG00000062146	AC119762.7	0.005636858	-1.723669375
ENSRNOG00000007792	Gcc1	0.005671368	1.455729029
ENSRNOG00000002142	Cds1	0.005675994	1.934551548
ENSRNOG00000001111	Brca2	0.005686904	-0.837190105
ENSRNOG00000000073	Tmed5	0.005713518	2.332953951
ENSRNOG00000011922	Stt3b	0.005719048	1.304676988
ENSRNOG00000020990	Fgf21	0.005770095	-1.80826333
ENSRNOG00000046183	AABR07055280.1	0.005804457	1.992074602
ENSRNOG00000015068	Il11ra1	0.005822012	-1.535723857
ENSRNOG00000043023	Msmp	0.005841967	1.93123427
ENSRNOG00000014235	Zgpat	0.005882095	1.512323506
ENSRNOG00000024886	Ext1	0.005979357	-0.948078213
ENSRNOG00000020350	Pacs1	0.006083781	2.116828893
ENSRNOG00000028569	Arhgap27	0.006106998	2.940537255
ENSRNOG00000007224	Bmt2	0.006112733	1.999176463
ENSRNOG00000024595	Cers6	0.006138612	1.242114324
ENSRNOG00000014723	Cbfa2t3	0.006144242	1.996783485
ENSRNOG00000017108	Syngr1	0.006153254	2.251100852
ENSRNOG00000026186	Syde2	0.006159019	2.179232449
ENSRNOG00000050111	Cenpo	0.006197678	2.281739581
ENSRNOG00000007354	Trpa1	0.006228173	1.71464622
ENSRNOG00000003562	Susd4	0.006265037	-0.880933815
ENSRNOG00000020743	Cyp2s1	0.006266586	1.109537821
ENSRNOG00000008354	Slc9a8	0.006286769	2.347381276
ENSRNOG00000012329	Saraf	0.006290825	0.836050614
ENSRNOG00000009113	Marcksl1	0.006299701	2.373201237
ENSRNOG00000020357	Msto1	0.006304075	2.611621384
ENSRNOG00000016885	Klf6	0.006319483	-0.898611902
ENSRNOG00000000665	Pitpnb	0.006326659	1.267235929
ENSRNOG00000056559	AABR07034669.1	0.006328786	1.777989551
ENSRNOG00000001351	Trafd1	0.006348245	-0.741195235

ENSRNOG00000018378	Cacnb2	0.006443364	1.212093949
ENSRNOG00000006718	Rbm33	0.006489957	1.903662673
ENSRNOG00000050437	Bod1l1	0.006509743	-1.211304677
ENSRNOG00000032009	#N/A	0.006528216	-2.360287713
ENSRNOG00000023053	Ice1	0.006540246	1.594705049
ENSRNOG00000004555	Sec14l4	0.006583825	2.122356915
ENSRNOG00000059720	Syp	0.006595334	1.999143123
ENSRNOG00000011431	Mfhas1	0.006629918	2.037674041
ENSRNOG00000015035	Myo7b	0.006679868	1.244041113
ENSRNOG00000015230	Ermard	0.006756243	-1.035509429
ENSRNOG00000008218	Atp6v0e2	0.006758934	1.583538217
ENSRNOG00000057622	Rbm41	0.006766174	-1.586847126
ENSRNOG00000032215	Cdyl	0.006776663	1.964951636
ENSRNOG00000051272	AABR07045350.1	0.006782068	-0.958321946
ENSRNOG00000000299	Foxo3	0.006784725	2.200441707
ENSRNOG00000037446	Pxmp2	0.006861504	-1.185785706
ENSRNOG00000010889	Fbxw7	0.006867379	-1.372726361
ENSRNOG00000054008	Scamp5	0.006891394	1.449564261
ENSRNOG00000015825	Aurkc	0.006975243	-0.840843242
ENSRNOG00000006327	Rcc2	0.006978786	2.397133489
ENSRNOG00000003268	Maml1	0.006995101	1.683163882
ENSRNOG00000028892	Prr36	0.007001177	1.593515216
ENSRNOG00000001335	Zkscan1	0.00701708	1.722245445
ENSRNOG00000060599	Gabrb3	0.007039986	1.31255322
ENSRNOG00000013774	Lmnb1	0.007108965	1.584048286
ENSRNOG00000012802	Tenm3	0.00716127	2.062554616
ENSRNOG00000054218	Il18rap	0.007199541	-1.560185397
ENSRNOG00000006353	#N/A	0.007224417	0.939683813
ENSRNOG00000017500	Mtss2	0.007239062	2.304167879
ENSRNOG00000001347	Adam1a	0.00727945	-0.933393198
ENSRNOG00000020087	Zfp90	0.007286469	-0.991877049
ENSRNOG00000018554	AABR07026797.1	0.007307586	1.390310034
ENSRNOG00000054557	Lsm14b	0.007348042	2.057893813
ENSRNOG00000004498	Scin	0.007368812	1.323351428
ENSRNOG00000049785	Ranbp3	0.007401286	2.174175803
ENSRNOG00000008424	Aagab	0.007466306	2.043266781
ENSRNOG00000046973	Tmco3	0.007475013	0.977960702
ENSRNOG00000033220	Oas1f	0.007479451	-0.981833684
ENSRNOG00000045918	#N/A	0.007497172	1.810471386
ENSRNOG00000030019	Atp1a1	0.007506331	1.093854569
ENSRNOG00000024241	Cdc23	0.007508154	1.431315563
ENSRNOG00000008075	Ift74	0.007551014	-1.331329529
ENSRNOG00000025184	Prss35	0.007557286	-2.379984546
ENSRNOG00000004377	Lpin1	0.007572935	2.072777072

ENSRNOG00000061763	AABR07012039.1	0.007579254	-3.158315886
ENSRNOG00000031665	Ace2	0.007581342	-1.850495441
ENSRNOG00000025216	Alkbh6	0.007624572	-1.75336666
ENSRNOG00000050655	P4ha1	0.007631117	0.937525854
ENSRNOG00000019189	Acat2	0.007632519	1.795476683
ENSRNOG00000022337	Slitrk6	0.007653364	-1.113232891
ENSRNOG00000020021	Brsk2	0.007681201	2.237202473
ENSRNOG00000004122	Kremen2	0.007728075	1.262295094
ENSRNOG00000016585	Bmi1	0.007731756	2.03349744
ENSRNOG00000027220	Pcdhgb8	0.007752006	2.066589881
ENSRNOG00000000873	Brs3	0.007756709	-2.27336946
ENSRNOG00000022288	Pafah2	0.007759828	1.899200656
ENSRNOG00000025164	Bhlha15	0.007783212	1.210662027
ENSRNOG00000056837	Abr	0.007968245	1.966114264
ENSRNOG00000004757	Tmem158	0.007976815	1.752243791
ENSRNOG00000001224	Itgb2	0.00798455	0.794171719
ENSRNOG00000014420	Rap2b	0.008003378	2.590744896
ENSRNOG00000019433	Rab3a	0.008011238	1.441026545
ENSRNOG00000052918	AABR07035802.1	0.008098178	-1.378458065
ENSRNOG00000054548	Srpk3	0.008152834	-1.078227745
ENSRNOG00000016245	Neto2	0.008179224	1.24579011
ENSRNOG00000029738	Diras1	0.008200128	1.960763305
ENSRNOG00000002818	Eftud2	0.008214644	1.520411907
ENSRNOG00000024470	#N/A	0.00822107	0.77045371
ENSRNOG00000028658	Olig2	0.008302012	1.675454762
ENSRNOG00000056213	AC130741.1	0.008326399	-1.275264477
ENSRNOG00000052096	Cox11	0.008334766	1.76858943
ENSRNOG00000019662	Tm6sf1	0.00836175	1.03995419
ENSRNOG00000024345	Pard3b	0.008386395	-1.563497248
ENSRNOG00000026914	Dnah1	0.008388079	-1.111813035
ENSRNOG00000052574	AABR07021955.1	0.008388179	1.517486664
ENSRNOG00000019697	Ankrd28	0.008388764	1.4449934
ENSRNOG00000018363	Hectd3	0.008515494	2.121242226
ENSRNOG00000000748	AABR07044273.1	0.00856318	2.13141558
ENSRNOG00000009746	Akap17b	0.00862552	-1.150758683
ENSRNOG00000029500	Tapbp	0.008635446	1.903041779
ENSRNOG00000029893	Rwdd3	0.008642392	-1.156884828
ENSRNOG00000052745	Met	0.008646738	1.518793194
ENSRNOG00000022623	Ttll12	0.008647635	1.969640757
ENSRNOG00000032178	Cenpa	0.008649953	1.791720084
ENSRNOG00000013642	Lrrc41	0.008653746	1.272705508
ENSRNOG00000030127	Eml2	0.008685751	1.26942128
ENSRNOG00000010457	Vash1	0.008714255	2.213054285
ENSRNOG00000048660	Haspin	0.008718148	0.954734703

ENSRNOG00000011624	Kcnq2	0.008746953	2.031607276
ENSRNOG00000012503	Dnajc16	0.008747668	2.459428172
ENSRNOG00000060395	AABR07025301.1	0.008768974	2.168918739
ENSRNOG00000008455	Tmem87a	0.008835358	1.248125873
ENSRNOG00000023520	Cs	0.008840331	1.327540777
ENSRNOG00000008372	Vamp7	0.008854629	1.017592434
ENSRNOG00000037199	Spink8	0.008878906	-1.447300547
ENSRNOG00000003326	Rrn3	0.008883634	1.099310749
ENSRNOG00000054614	AABR07002779.1	0.008946151	2.268462421
ENSRNOG00000055936	Trnp1	0.008960791	2.820837091
ENSRNOG00000048431	Nynrin	0.008978773	2.865532424
ENSRNOG00000050547	Syngr2	0.009013413	1.570940487
ENSRNOG00000025757	Myh6	0.009041974	1.975118269
ENSRNOG00000057934	Tmx1	0.009054889	0.949004671
ENSRNOG00000028215	Nup160	0.00905685	1.845675185
ENSRNOG00000014834	#N/A	0.009101552	1.304315802
ENSRNOG00000011402	Isg20l2	0.009110229	1.388429012
ENSRNOG00000009724	Tstd2	0.009191881	1.693558878
ENSRNOG00000007418	Ipo9	0.009199896	1.422431014
ENSRNOG00000002246	Slc30a9	0.009200867	1.117156954
ENSRNOG00000012878	Atp7b	0.00921022	2.115966155
ENSRNOG00000016818	Fgfr3	0.009253959	1.874148464
ENSRNOG00000007246	Atxn7	0.009276575	1.69520119
ENSRNOG00000005434	Sptbn1	0.009293645	1.744058765
ENSRNOG00000006298	Cpne3	0.009305012	1.288781403
ENSRNOG00000020014	Myh14	0.009396984	1.858253346
ENSRNOG00000019063	Fbxo38	0.009402568	0.950307941
ENSRNOG00000002546	Rchy1	0.009430644	0.981121517
ENSRNOG00000050500	Tob2	0.009462888	1.900243089
ENSRNOG00000007189	Ttc22	0.009487236	1.45254243
ENSRNOG00000014233	Krt19	0.009499562	2.075323147
ENSRNOG00000013570	Rad54l2	0.009507798	1.811715219
ENSRNOG00000001474	Tmem270	0.009534908	-1.175278702
ENSRNOG00000007054	Dhx33	0.0096096	2.544920617
ENSRNOG00000010176	Map2k1	0.009612203	0.814326605
ENSRNOG00000025864	LOC108348120	0.009616925	2.323868143
ENSRNOG00000008652	Phip	0.009656696	-1.860871628
ENSRNOG00000007512	Srp14	0.009667708	2.737755471
ENSRNOG00000020532	Kcnq1	0.00969657	1.442587331
ENSRNOG00000010827	Ptbp2	0.009713187	1.288941439
ENSRNOG00000049334	Atp2c2	0.009763571	1.364228536
ENSRNOG00000005276	Csnk2a1	0.00977574	1.448557224
ENSRNOG00000046445	Rcor3	0.009845157	2.164530825
ENSRNOG00000001164	Tff1	0.00989041	-1.243622819

ENSRNOG00000046460	AABR07022144.1	0.009893744	-1.120076409
ENSRNOG00000013973	Lcn2	0.009897614	-1.498932292
ENSRNOG00000015878	Pif1	0.009928161	2.763017139
ENSRNOG00000000728	Clic2	0.010085506	2.155520362
ENSRNOG00000011290	Tm2d3	0.010141407	0.998605131
ENSRNOG00000027151	Lrrc58	0.010159441	2.249239931
ENSRNOG00000012346	Epb41l2	0.010166221	1.954561601
ENSRNOG00000046500	Irgq	0.010187866	2.693791498
ENSRNOG00000017032	Atp5f1a	0.010225054	0.896099379
ENSRNOG00000005413	Creb3l1	0.010252458	0.867211929
ENSRNOG00000031285	AABR07064305.1	0.0103383	1.195142499
ENSRNOG00000039976	Ptk7	0.010338587	2.540241667
ENSRNOG00000032048	Zfp462	0.010501978	0.834822217
ENSRNOG00000029799	Tomm34	0.010504427	0.865415554
ENSRNOG00000013679	Sema4d	0.010545213	1.81705654
ENSRNOG00000021902	Rmi2	0.010563396	2.776167354
ENSRNOG00000001083	Kdelr2	0.010620713	0.800305258
ENSRNOG00000021118	Nomo1	0.010677706	0.948850908
ENSRNOG00000014343	Anln	0.010717947	1.594677988
ENSRNOG00000023410	Apol9a	0.010758354	-0.883700361
ENSRNOG00000011509	Agk	0.010759006	1.383308892
ENSRNOG00000008616	Ubr3	0.010762679	1.265263574
ENSRNOG00000059362	Has3	0.010778285	-0.899073421
ENSRNOG00000028063	Tmem38b	0.01093001	0.822040738
ENSRNOG00000003929	Pcdh19	0.010947552	-1.248034974
ENSRNOG00000010697	Hadh	0.01095219	1.420347786
ENSRNOG00000045740	Tmx3	0.010995067	1.194768979
ENSRNOG00000031590	AABR07048308.2	0.010997852	1.302703151
ENSRNOG00000005165	Smcr8	0.011027388	1.931659509
ENSRNOG00000028208	Mief2	0.011038142	2.390211578
ENSRNOG00000047799	Gnb5	0.011070509	1.664440963
ENSRNOG00000026171	Bbs4	0.011122396	-0.836145361
ENSRNOG00000032522	B630019K06Rik	0.011158757	2.363143181
ENSRNOG00000055391	Eif4ebp2	0.011162277	1.616761389
ENSRNOG00000025580	LOC100912618	0.011195305	1.319386292
ENSRNOG00000013802	Slc25a36	0.011236353	1.511582714
ENSRNOG00000045920	Mcu	0.011290567	1.449027333
ENSRNOG00000013196	Dok5	0.011302077	0.906693223
ENSRNOG00000009419	Ptprg	0.011332174	1.883278513
ENSRNOG00000060936	Ikbkg	0.011337257	1.971384688
ENSRNOG00000017856	Fev	0.011381675	-0.780528525
ENSRNOG00000014872	Sec24d	0.011419564	1.105313064
ENSRNOG00000020080	Lrrc18	0.011457011	-2.292124412
ENSRNOG00000012356	Slc36a1	0.011497467	1.818499691

ENSRNOG00000018873	Fam168a	0.011511457	1.529292416
ENSRNOG00000001823	St6gal1	0.011525546	-0.892538579
ENSRNOG00000030180	Lrrc10b	0.011563748	1.994087004
ENSRNOG00000018840	Rnf40	0.011579874	1.980906844
ENSRNOG00000015735	Zswim1	0.01159182	1.488671647
ENSRNOG00000001198	Fam222a	0.011610438	2.768227052
ENSRNOG00000033101	Myo18a	0.011640546	1.794503077
ENSRNOG00000006003	Ctnna2	0.011647533	1.664581046
ENSRNOG00000054907	AABR07012100.2	0.011732646	-2.219373233
ENSRNOG00000055281	Dcbld2	0.01181788	1.503313078
ENSRNOG00000033734	Tnnt2	0.011867274	1.757776585
ENSRNOG00000020875	Celf3	0.011904832	1.789829562
ENSRNOG00000010208	Timp1	0.011920739	-1.57621572
ENSRNOG00000014241	Ece1	0.011933655	1.731096416
ENSRNOG00000002680	Lamc1	0.011999716	1.515363247
ENSRNOG00000011511	Stk24	0.011999995	1.712415247
ENSRNOG00000028534	Zfp518b	0.012015363	2.197828105
ENSRNOG00000018798	Bcan	0.012049879	1.999354706
ENSRNOG00000020095	Pcdhb3	0.012066707	1.302150175
ENSRNOG00000028611	AABR07037203.1	0.012187529	0.897516153
ENSRNOG00000024201	Fer1l5	0.012203587	-0.879581496
ENSRNOG00000012002	Iqgap1	0.012219725	1.439888433
ENSRNOG00000001868	LOC100911248	0.012288068	1.590530252
ENSRNOG00000059683	Mpp2	0.012291452	2.517568109
ENSRNOG00000003614	Mgat5	0.012327224	1.702756498
ENSRNOG00000048462	AABR07066529.1	0.012332322	1.736235156
ENSRNOG00000001582	Bach1	0.012376697	2.572934557
ENSRNOG00000000529	Pim1	0.01239817	2.592263966
ENSRNOG00000024482	Tnrc18	0.012398951	2.130458028
ENSRNOG00000015501	Ddhd2	0.012430283	1.593116609
ENSRNOG00000004217	Stk10	0.012507196	2.449656922
ENSRNOG00000007906	Bub1b	0.0126551	1.192143037
ENSRNOG00000029778	Maob	0.012659606	1.2459836
ENSRNOG00000023400	Dtx3l	0.012722937	1.506867304
ENSRNOG00000016050	Fgfr1	0.012746675	1.850983088
ENSRNOG00000030704	AC094053.1	0.012861066	1.413993919
ENSRNOG00000017225	Fam160b1	0.012899447	1.750195235
ENSRNOG00000048929	AABR07044389.1	0.012908626	2.477529426
ENSRNOG00000014844	Kif21a	0.012937815	1.443238913
ENSRNOG00000008244	LOC690035	0.012942349	-0.810941042
ENSRNOG00000057754	Tcf12	0.013003102	1.94306648
ENSRNOG00000006787	Dhcr24	0.013016124	1.226792271
ENSRNOG00000002524	Gpr37	0.013028535	1.064747929
ENSRNOG00000018958	Mt3	0.013033614	-0.864277411

ENSRNOG00000042326	Smpdl3b	0.013140544	1.964907176
ENSRNOG00000014505	Pmfbp1	0.013160993	-1.741014884
ENSRNOG00000008837	Ass1	0.013204074	0.931256891
ENSRNOG00000006770	Brd4	0.013222104	-1.080027541
ENSRNOG00000048145	Sstr1	0.013284253	1.346177789
ENSRNOG00000060775	Lmo7	0.013352057	1.24631721
ENSRNOG00000013441	Gnrh1	0.013374639	-1.566271993
ENSRNOG00000060146	Six5	0.013375502	2.652442215
ENSRNOG00000006889	Ambp	0.013394501	-0.797304163
ENSRNOG00000062252	AABR07072853.5	0.013602614	1.472675992
ENSRNOG00000009163	Fam133b	0.013629749	-1.328247486
ENSRNOG00000003551	B4galt3	0.013652719	0.985669575
ENSRNOG00000058662	Ano8	0.013657424	2.332803314
ENSRNOG00000008145	Traf3	0.013660225	1.57853888
ENSRNOG00000030579	AC126572.2	0.013692426	2.699271289
ENSRNOG00000049162	Prr15l	0.0137219	3.045948903
ENSRNOG00000024813	Mzt1	0.013743452	2.463393962
ENSRNOG00000014186	Zswim9	0.013784732	1.348059528
ENSRNOG00000001252	Chst12	0.013832373	1.268433167
ENSRNOG00000008291	Thumpd2	0.013848367	-0.789967262
ENSRNOG00000020873	Nphs1	0.013865861	-1.830760051
ENSRNOG00000004263	Ints7	0.013871904	1.141846808
ENSRNOG00000052386	Nlrx1	0.01390071	1.92439862
ENSRNOG00000001593	Lnpk	0.013910456	0.90203097
ENSRNOG00000007315	Thoc2	0.013934089	-1.621909124
ENSRNOG00000059962	AABR07025787.1	0.013961983	1.206627834
ENSRNOG00000000064	Atp5me	0.013977579	-0.875379497
ENSRNOG00000003049	Hsd17b10	0.014014848	2.33096684
ENSRNOG00000030149	Adgrl3	0.014021726	1.065373479
ENSRNOG00000000145	Pik3r3	0.014038218	1.481931176
ENSRNOG00000000657	Nek7	0.014091962	1.969682285
ENSRNOG00000000483	Syngap1	0.014104391	2.359111418
ENSRNOG00000024213	Golim4	0.014135517	1.659572197
ENSRNOG00000005902	Ccdc34	0.014179171	-1.551409257
ENSRNOG00000005814	NEWGENE_1582771	0.014201395	1.139343854
ENSRNOG00000020694	Icam5	0.014206173	1.878845703
ENSRNOG00000020435	Terf2	0.014224193	1.228537637
ENSRNOG00000013930	Rnf4	0.014305401	1.048266095
ENSRNOG00000020663	Pygo2	0.014316487	1.135003409
ENSRNOG00000062283	AABR07003492.2	0.014366254	2.186516836
ENSRNOG00000061434	AC128293.1	0.014396141	-1.781726386
ENSRNOG00000019298	Dctn4	0.014418815	1.572904162
ENSRNOG00000003948	Llgl1	0.014436456	1.95962371
ENSRNOG00000052140	Ogg1	0.014444855	1.144093318

ENSRNOG00000008625	Rimkla	0.014472968	1.324551247
ENSRNOG00000059094	U6	0.014486377	-3.21337906
ENSRNOG00000005916	Ppm1a	0.014501313	0.789261482
ENSRNOG00000001342	Wdr66	0.014557951	-0.946974486
ENSRNOG00000020349	Rab3il1	0.014578203	1.916315939
ENSRNOG00000046790	AABR07040887.1	0.014738223	-1.225589621
ENSRNOG00000018358	Nt5dc2	0.014803934	1.146450996
ENSRNOG00000008706	Tbx3	0.014860285	1.50967363
ENSRNOG00000059116	AABR07035818.1	0.014931348	1.501770973
ENSRNOG00000030830	#N/A	0.014952467	0.997922359
ENSRNOG00000056040	AABR07045680.1	0.014952732	-1.274676881
ENSRNOG00000048843	Tsfm	0.015040138	1.636780539
ENSRNOG00000046905	Sgce	0.015065679	1.247182194
ENSRNOG00000026277	Zc3h6	0.015070226	-1.052120844
ENSRNOG00000020424	Plpp4	0.015074198	-0.961426126
ENSRNOG00000013323	Rnf217	0.015085733	1.206536178
ENSRNOG00000020478	Camk4	0.015088864	-1.122023099
ENSRNOG00000032813	Mdc1	0.015097262	1.702168208
ENSRNOG00000024243	Cadm4	0.015114509	2.134266633
ENSRNOG00000031974	Pcdh10	0.015166069	1.662307717
ENSRNOG00000029304	Aggf1	0.015267032	1.313062402
ENSRNOG00000015576	Hsdl1	0.015317833	1.460383079
ENSRNOG00000013844	Fam172a	0.015324814	2.051358586
ENSRNOG00000008862	Abcg4	0.015344805	2.587130307
ENSRNOG00000060359	AABR07032751.1	0.015349082	1.524362933
ENSRNOG00000013670	Pax3	0.015379728	2.398963444
ENSRNOG00000031544	Larp1	0.015389069	1.502844569
ENSRNOG00000010497	RGD1305807	0.015390353	-0.754043156
ENSRNOG00000032590	Ttc28	0.015445342	-1.003558021
ENSRNOG00000046207	Cbx4	0.01550074	1.983416633
ENSRNOG00000014785	Ykt6	0.015500948	0.902078239
ENSRNOG00000062168	AC119762.8	0.015628487	-1.390064796
ENSRNOG00000059374	AABR07024457.1	0.015664767	-2.250995634
ENSRNOG00000020860	Tdrkh	0.015671309	-0.781585564
ENSRNOG00000003759	Galc	0.015752337	1.530303197
ENSRNOG00000020417	Gsk3a	0.01575242	1.294356373
ENSRNOG00000020466	Apba3	0.015850765	2.292232792
ENSRNOG00000001250	Lfng	0.015892881	1.467390423
ENSRNOG00000012333	Kbtbd11	0.015908953	1.626535099
ENSRNOG00000024905	Drc1	0.015912186	-0.753376041
ENSRNOG00000029726	Gstm1	0.015955713	-1.156656817
ENSRNOG00000047454	AABR07054716.1	0.015959715	-1.70872617
ENSRNOG00000011975	Crtc3	0.015990048	1.828729407
ENSRNOG00000011476	Nars2	0.015995498	1.397291335

ENSRNOG00000007725	Mis12	0.01607649	1.722984033
ENSRNOG00000047734	Chst2	0.016125047	1.545914913
ENSRNOG00000018690	Rgs17	0.016173617	-1.005051753
ENSRNOG00000014851	Col4a4	0.016187679	-0.934167276
ENSRNOG00000012629	Rab23	0.016208374	0.989099592
ENSRNOG00000014712	Zfp39	0.016252575	-1.898772991
ENSRNOG00000016246	Tshz1	0.016273128	2.085159109
ENSRNOG00000016254	Sema4c	0.016320749	0.875885186
ENSRNOG00000032183	Scmh1	0.016348914	1.65918577
ENSRNOG00000016935	Mapk1ip1	0.016364983	-0.872140905
ENSRNOG00000049033	Racgap1	0.016370824	1.237088207
ENSRNOG00000028279	Tex52	0.016425107	-0.949303261
ENSRNOG00000043099	Ddx21	0.016475531	1.076309267
ENSRNOG00000023816	Aph1a	0.016489662	0.873777585
ENSRNOG00000009152	Caprin1	0.016512	0.784637484
ENSRNOG00000023226	S100a10	0.016602971	-0.983464266
ENSRNOG00000056941	AABR07066529.3	0.016637951	-1.008963712
ENSRNOG00000026458	Tbccd1	0.016697352	1.452167188
ENSRNOG00000008622	Creb5	0.016777384	-2.186153061
ENSRNOG00000001255	Mlxip	0.01677786	1.755970882
ENSRNOG00000011847	Grk4	0.016849014	-0.911046926
ENSRNOG00000055597	Hdac7	0.016862322	2.349277531
ENSRNOG00000011157	Jak1	0.016868326	0.953380583
ENSRNOG00000034233	Fyttd1	0.016890705	1.117149651
ENSRNOG00000058156	Zdhhc18	0.016909383	1.559782014
ENSRNOG00000018646	Hbegf	0.016917265	-0.831000873
ENSRNOG00000019518	Pde4c	0.016929031	1.879520358
ENSRNOG00000023465	Depp1	0.016955797	-1.649597225
ENSRNOG00000005438	Pcsk2	0.016980767	0.773580928
ENSRNOG00000015124	Gpam	0.017002554	1.727836525
ENSRNOG00000025060	Nanos1	0.017012209	1.953640021
ENSRNOG00000000500	Scube3	0.017025783	-1.1846144
ENSRNOG00000019482	Gnao1	0.017042861	1.110390836
ENSRNOG00000027770	Trpm3	0.017048531	1.23639787
ENSRNOG00000043364	Zfp362	0.017103726	1.694028583
ENSRNOG00000047873	Sec22c	0.017103743	1.627261848
ENSRNOG00000058663	Suz12	0.017164749	1.302891644
ENSRNOG00000030247	Eif4ebp3	0.017171043	-1.024975715
ENSRNOG00000002930	Ppl	0.01718136	0.957254289
ENSRNOG00000018111	Slc12a5	0.017187987	2.081469607
ENSRNOG00000011857	Mtpn	0.017191112	1.234102462
ENSRNOG00000003576	Ints2	0.017253833	1.479499325
ENSRNOG00000054542	#N/A	0.017263789	-1.885400166
ENSRNOG00000039207	Ccdc71l	0.017294243	2.365122766

ENSRNOG00000017156	Ankle1	0.017327065	2.058969475
ENSRNOG00000030871	Calm2	0.017343762	0.91908706
ENSRNOG00000020996	Vps51	0.01734965	2.042026849
ENSRNOG00000004956	Jade2	0.017383772	2.550190378
ENSRNOG00000049281	Gba	0.017430488	1.423692977
ENSRNOG00000061488	AABR07026557.2	0.017438811	-1.791745145
ENSRNOG00000007219	LOC103692716	0.017654982	-1.923476462
ENSRNOG00000025423	Map3k13	0.017656853	1.115868685
ENSRNOG00000004027	Aldh9a1	0.017741243	1.253009982
ENSRNOG00000028844	Slc9a5	0.017765308	0.973715547
ENSRNOG00000017690	Ccsap	0.017849393	2.179554903
ENSRNOG00000029244	Pcdhb10	0.017853017	0.928431824
ENSRNOG00000043037	Zfp770	0.017890433	1.68106525
ENSRNOG00000003398	Tomm40l	0.017897854	1.744698204
ENSRNOG00000002873	Fam183a	0.017957116	-0.974260921
ENSRNOG00000021437	AABR07073181.1	0.017962617	2.525296278
ENSRNOG00000019843	St3gal3	0.018030664	1.893153668
ENSRNOG00000012269	Lpcat3	0.018031901	1.046440914
ENSRNOG00000008960	#N/A	0.018043166	1.671092246
ENSRNOG00000007410	Dab1	0.018064292	-0.905642409
ENSRNOG00000010104	Pofut1	0.018073043	1.613457738
ENSRNOG00000029082	AABR07005031.1	0.018106443	1.31090734
ENSRNOG00000009439	Eef1a1	0.01819182	0.86578037
ENSRNOG00000025818	Zxdc	0.018221883	1.538184326
ENSRNOG00000033262	Reep6	0.018224216	0.866909806
ENSRNOG00000060905	AC111831.2	0.01822736	2.408386009
ENSRNOG00000011910	Hnrnpr	0.018241925	0.904929514
ENSRNOG00000053078	Rnu11	0.018285762	-1.594998037
ENSRNOG00000008401	Card10	0.018300268	2.640206123
ENSRNOG00000025551	Rgs22	0.018396345	-0.85404595
ENSRNOG00000050535	RragB	0.018422605	-2.77952264
ENSRNOG00000002835	Luc7l3	0.018426232	-1.337355654
ENSRNOG00000059702	Tex9	0.018573065	-1.231771483
ENSRNOG00000011238	Tiparp	0.018575028	1.667223141
ENSRNOG00000021150	Plcb3	0.018633316	1.652564778
ENSRNOG00000047247	Ptprs	0.018692898	1.885324083
ENSRNOG00000022421	Crtc1	0.018693165	1.558992226
ENSRNOG00000011161	Slc2a12	0.018695015	-1.528039245
ENSRNOG00000002710	#N/A	0.018704669	2.010460963
ENSRNOG00000039876	LOC681410	0.018704768	1.96718736
ENSRNOG00000059017	AC106292.2	0.018749958	1.655316806
ENSRNOG00000000155	Cog3	0.018776401	1.550946127
ENSRNOG00000010517	Fam126a	0.01882167	1.620156313
ENSRNOG00000005108	Wfs1	0.018912729	0.892038888

ENSRNOG00000014180	Prickle4	0.018931564	2.059741281
ENSRNOG00000014686	Kcnd3	0.01895346	-1.717953737
ENSRNOG00000007371	Rbmx2	0.01896021	-1.049723951
ENSRNOG00000011953	Supt16h	0.018984703	0.835535241
ENSRNOG00000014503	Celf4	0.018999716	1.440100186
ENSRNOG00000010533	Arfip1	0.019014138	0.890900154
ENSRNOG00000002883	Alg1	0.019126823	1.598113078
ENSRNOG00000021578	#N/A	0.019150237	-0.884784904
ENSRNOG00000026344	Togaram2	0.019264704	-0.791002553
ENSRNOG00000048951	LOC100364500	0.019277825	1.8244539
ENSRNOG00000047367	Card14	0.01933354	1.827305622
ENSRNOG00000013581	Extl3	0.019340631	1.782165742
ENSRNOG00000025792	Crk	0.019382624	1.464811489
ENSRNOG00000061206	AABR07064618.1	0.019420466	-3.336237532
ENSRNOG00000028586	Mcph1	0.019430555	1.62565295
ENSRNOG00000055222	AC094212.1	0.01943477	1.452485673
ENSRNOG00000006952	Prex1	0.019668653	2.289200159
ENSRNOG00000009482	Emx2	0.019724111	1.947172186
ENSRNOG00000008490	Cwc15	0.019754941	-0.89346429
ENSRNOG00000000397	Ccar1	0.019773993	-1.237102235
ENSRNOG00000016544	Arhgef28	0.019797515	0.782382781
ENSRNOG00000057557	Prlr	0.019836134	1.286913875
ENSRNOG00000002941	Uhmk1	0.019855401	1.120194335
ENSRNOG00000010591	Kdm5a	0.019908688	-1.134456795
ENSRNOG00000010434	Dync1li1	0.019965489	1.389979734
ENSRNOG00000009040	Gas2l1	0.019967383	3.604169693
ENSRNOG00000002488	#N/A	0.019989675	1.814760977
ENSRNOG00000006711	Zfp212	0.020063074	1.968697907
ENSRNOG00000034200	Atp8a1	0.020075326	1.442619629
ENSRNOG00000015664	Tmem8b	0.020091405	1.698460007
ENSRNOG00000027867	Rexo4	0.020106688	0.860297859
ENSRNOG00000053210	Zc3h11a	0.020108942	1.688692647
ENSRNOG00000015397	Cpne7	0.020145924	-0.742443979
ENSRNOG00000010119	Zmat3	0.020158649	1.594243583
ENSRNOG00000011254	Fam76a	0.020188128	2.067422793
ENSRNOG00000043093	Ap1m2	0.020237298	0.788030038
ENSRNOG00000048978	Nphp3	0.020293876	1.882237315
ENSRNOG00000056017	Bcl7a	0.020297835	2.468275756
ENSRNOG00000061237	Dio1	0.020310798	-1.886307922
ENSRNOG00000055939	Zcchc18	0.020338781	1.018174931
ENSRNOG00000020044	Mob2	0.020394346	1.008216377
ENSRNOG00000047295	Prr22	0.020415318	-0.828881956
ENSRNOG00000006033	#N/A	0.020419931	-0.774724464
ENSRNOG00000005715	Lgr4	0.02042358	1.076652567

ENSRNOG00000047739	Npff	0.020478589	-1.132617444
ENSRNOG00000004402	Lpgat1	0.02051468	1.093333075
ENSRNOG00000012237	Rdx	0.020522074	-0.991532907
ENSRNOG00000026502	Dscc1	0.020571967	-0.85685202
ENSRNOG00000015511	Atic	0.020581447	1.006293361
ENSRNOG00000011823	Tfap2b	0.020589623	-1.076962983
ENSRNOG00000048004	Garem2	0.020654353	2.374941866
ENSRNOG00000055499	Grid1	0.020676312	1.496879731
ENSRNOG00000046023	Exoc4	0.020683858	1.631222429
ENSRNOG00000002882	Zcchc2	0.020704056	1.417124243
ENSRNOG00000022298	Cxcl11	0.020711726	-1.7694749
ENSRNOG00000027469	Pcdhb4	0.020714064	1.129639516
ENSRNOG00000018986	Fbxl19	0.020763065	1.359193131
ENSRNOG00000009157	Fut4	0.020851818	0.808280829
ENSRNOG00000060120	Selenoo	0.02088025	1.890010714
ENSRNOG00000031485	#N/A	0.020989869	1.758422645
ENSRNOG00000055096	AABR07002768.1	0.021023388	-1.639093385
ENSRNOG00000011339	Slk	0.021063513	1.166911439
ENSRNOG00000028908	Eppin	0.021093878	1.704441279
ENSRNOG00000060105	Astn2	0.021094472	1.716196022
ENSRNOG00000008421	Klhl5	0.021095602	1.858113616
ENSRNOG00000042464	Fbxo25	0.021103427	1.202759213
ENSRNOG00000005775	Phf14	0.021106253	-0.819703065
ENSRNOG00000013912	Slc30a7	0.021122239	0.766588309
ENSRNOG00000003508	#N/A	0.021127394	1.704156673
ENSRNOG00000019830	Hsd17b1	0.021203503	-0.788097804
ENSRNOG00000008513	LOC103690019	0.02122136	2.674710498
ENSRNOG00000018319	Pisd	0.021227421	1.794957346
ENSRNOG00000029264	Kcnn1	0.021237393	1.603445282
ENSRNOG00000004473	Ppargc1a	0.021244642	1.038913395
ENSRNOG00000019500	Cyp1a1	0.021249843	-1.287930214
ENSRNOG00000002001	Itsn1	0.021251661	1.738317996
ENSRNOG00000011313	Sorcs1	0.021267635	2.115398644
ENSRNOG00000003873	Cpd	0.021290476	1.392435959
ENSRNOG00000009863	Prrt3	0.02133383	2.409698011
ENSRNOG00000053099	Wfdc15a	0.021451378	-2.13784318
ENSRNOG00000060312	LOC108348197	0.021471883	1.646436536
ENSRNOG00000009180	Xkr7	0.0214929	1.783743003
ENSRNOG00000009207	Spata2	0.02157216	1.829497481
ENSRNOG00000018485	Gpr155	0.021706398	0.801983396
ENSRNOG00000027574	AABR07053879.1	0.021707676	1.386466671
ENSRNOG00000003721	Paqr4	0.021781068	1.987632003
ENSRNOG00000004978	Prkacb	0.021787507	2.011880187
ENSRNOG00000033776	LOC100359668	0.021821251	1.912884639

ENSRNOG00000033490	Vsig2	0.021905014	1.201424623
ENSRNOG00000024372	Cwf19l2	0.021911582	-1.445373519
ENSRNOG00000022971	#N/A	0.021927294	-1.270563912
ENSRNOG00000000456	Psmb8	0.021967069	1.513912957
ENSRNOG00000015468	Bpifb1	0.021975698	-1.196351307
ENSRNOG00000032847	AABR07060145.1	0.022010518	-1.127735191
ENSRNOG00000008831	Hcn2	0.022072846	1.87367868
ENSRNOG00000036960	Abcc9	0.022075403	-1.689022173
ENSRNOG00000008088	Btbd3	0.022108233	1.125382097
ENSRNOG00000048898	Wasf3	0.022135635	1.4955064
ENSRNOG00000012148	Trio	0.02225888	1.832186715
ENSRNOG00000023348	Tbc1d2	0.022380412	1.669822585
ENSRNOG00000032391	Esyt2	0.022410169	1.564049587
ENSRNOG00000018952	Sema3g	0.022441342	2.139924285
ENSRNOG00000059688	AABR07030377.1	0.022514542	-2.718497841
ENSRNOG00000037765	Lims1	0.022583307	1.89561271
ENSRNOG00000001767	RGD1562339	0.022612457	2.16565985
ENSRNOG00000015830	Ano9	0.022679882	1.468106832
ENSRNOG00000024757	AABR07069219.1	0.022735059	1.159825389
ENSRNOG00000021017	Ca11	0.022742655	-0.802528132
ENSRNOG00000015236	Mybbp1a	0.022751011	1.401186544
ENSRNOG00000025704	Yod1	0.022758675	1.755766651
ENSRNOG00000033090	Ltbp1	0.022799418	-0.842283073
ENSRNOG00000049452	Nebl	0.022858576	-0.811325486
ENSRNOG00000018764	B3gnt3	0.022886523	1.075211843
ENSRNOG00000013503	Cdh24	0.023065103	1.612483952
ENSRNOG00000019014	Ndst1	0.023070341	1.610718845
ENSRNOG00000057125	Ddr1	0.023144931	0.783758416
ENSRNOG00000004032	Tmem94	0.023162174	1.500008253
ENSRNOG00000001773	Senp2	0.023184007	1.189444405
ENSRNOG00000038002	Ccdc62	0.023199516	-1.019786993
ENSRNOG00000023151	Scgb2a1	0.023242755	-2.194565673
ENSRNOG00000023317	Colgalt1	0.023247005	1.122172756
ENSRNOG00000025648	Dhrs7l1	0.023309325	1.714182985
ENSRNOG00000005472	Sp4	0.023353347	2.201961721
ENSRNOG00000057382	U1	0.023376516	-2.403533621
ENSRNOG00000008689	Cbx1	0.023431751	1.213327065
ENSRNOG00000054461	RNaseP_nuc	0.023445207	-2.505861468
ENSRNOG00000018823	Nisch	0.023483179	1.704733352
ENSRNOG00000024651	Greb1	0.023530163	-1.800293444
ENSRNOG00000002289	Ildr1	0.023533769	1.188677082
ENSRNOG00000008474	Acox3	0.023558423	1.986682018
ENSRNOG00000003287	Fbxw10	0.023576319	-2.072249517
ENSRNOG00000026091	Slc10a4	0.023655571	0.864364234

ENSRNOG00000018145	Crat	0.023691104	1.368462447
ENSRNOG00000013318	Ccdc150	0.023698914	-1.198010355
ENSRNOG00000003218	Zfp286a	0.023732271	-1.052671664
ENSRNOG00000020653	S1pr2	0.023773387	0.837057857
ENSRNOG00000004476	Wif1	0.02379126	1.103167838
ENSRNOG00000013618	Ankrd10	0.02380427	1.681778908
ENSRNOG00000031045	LOC100911252	0.023914671	1.044880925
ENSRNOG00000013728	Polg2	0.023950156	-0.787766996
ENSRNOG00000012950	Efr3b	0.023987011	1.927182838
ENSRNOG00000019948	Phf20	0.024007042	-0.900520786
ENSRNOG00000003840	Slit2	0.024049859	-0.895316064
ENSRNOG00000022143	Dusp23	0.024257628	1.845329568
ENSRNOG00000004753	Napb	0.024259512	1.454550935
ENSRNOG00000022260	Senp1	0.024312589	1.553379772
ENSRNOG00000050657	Crlf3	0.024444349	1.203618686
ENSRNOG00000005673	Runx1t1	0.024646134	-1.425986767
ENSRNOG00000053067	AABR07035008.1	0.024654669	-1.801906163
ENSRNOG00000022505	Slc17a4	0.024786663	-1.322000317
ENSRNOG00000047780	AABR07046635.1	0.024796593	-2.285620917
ENSRNOG00000049531	Usp19	0.024798361	1.340880525
ENSRNOG00000018522	LOC100911186	0.024821998	1.625460194
ENSRNOG00000028052	RGD1563620	0.024831934	1.829486389
ENSRNOG00000018020	Apbb1	0.024926024	1.197459843
ENSRNOG00000000795	RT1-N3	0.024947503	2.170952474
ENSRNOG00000019027	Habp4	0.025116084	1.29408029
ENSRNOG00000007115	Crebl2	0.025132782	1.759201195
ENSRNOG00000024172	T2	0.02523289	-1.038278299
ENSRNOG00000037302	Rad51	0.025249241	1.325316225
ENSRNOG00000014478	Fndc3a	0.02536498	1.078960819
ENSRNOG00000014766	Galt	0.025387362	-0.773037058
ENSRNOG00000013517	Phtf2	0.025505553	-0.766727908
ENSRNOG00000014714	Trpv6	0.025529064	-0.756086181
ENSRNOG00000004709	Foxn3	0.025737347	-1.098402788
ENSRNOG00000024349	Cbarp	0.025759999	1.840483262
ENSRNOG00000020539	Muc1	0.02577441	-1.003609878
ENSRNOG00000023814	Rimklb	0.025846888	1.121348133
ENSRNOG00000010608	Cep162	0.025853499	-0.914498999
ENSRNOG00000007916	Ptk2	0.025865639	1.184034874
ENSRNOG00000002196	Ociad2	0.025883837	1.512866916
ENSRNOG00000016210	Micalcl	0.026009096	-0.761572279
ENSRNOG00000004608	Pam16	0.02606611	-0.806568704
ENSRNOG00000046969	AABR07034438.1	0.026149519	1.26177237
ENSRNOG00000061543	Ap2b1	0.026168296	1.149831809
ENSRNOG00000017707	Abi2	0.026184886	1.506842406

ENSRNOG00000010379	Celf1	0.026193925	0.841151351
ENSRNOG00000015914	U2af2	0.026198229	1.358394337
ENSRNOG00000023021	Msl2	0.026329041	1.01806309
ENSRNOG00000014152	Kcnip3	0.026363096	1.587551319
ENSRNOG00000028628	LOC100911196	0.026504746	1.509979416
ENSRNOG00000056678	Nckap5l	0.026599486	1.633461196
ENSRNOG00000052814	Ankrd27	0.026624136	1.379057878
ENSRNOG00000049088	Ttc32	0.026625735	-1.237412884
ENSRNOG00000016269	Rpl7l1	0.026639227	1.408346982
ENSRNOG00000010896	Tprn	0.026694695	0.893595563
ENSRNOG00000012534	Mfsd10	0.026775871	1.232089431
ENSRNOG00000015409	Usp5	0.026921665	1.250523208
ENSRNOG00000049523	AC097153.2	0.026925481	-2.578810502
ENSRNOG00000015380	Jup	0.027024906	0.793996898
ENSRNOG00000038184	Camk2n2	0.027027885	3.332756351
ENSRNOG00000025594	Scrt1	0.027064687	2.302171544
ENSRNOG00000055694	LOC102553190	0.027072988	-1.054877977
ENSRNOG00000052357	Fosl2	0.027178831	2.494107007
ENSRNOG00000004414	F11r	0.027222927	1.190641864
ENSRNOG00000032546	Dot1l	0.027237783	1.375833091
ENSRNOG00000023837	Mtx3	0.027256621	1.007467453
ENSRNOG00000051085	AABR07011062.1	0.027304432	-2.277478005
ENSRNOG00000023614	Hsh2d	0.027341798	1.365383946
ENSRNOG00000000662	#N/A	0.027380043	1.526893067
ENSRNOG00000002192	Rell1	0.027418981	0.851920341
ENSRNOG00000003649	Qsox1	0.027422939	1.146453245
ENSRNOG00000018400	Golm1	0.027484412	1.364042566
ENSRNOG00000029377	Rpl38	0.027610398	-1.589971329
ENSRNOG00000010194	Ift43	0.027644278	-0.745984096
ENSRNOG00000009598	Ncaph2	0.027704397	1.462941046
ENSRNOG00000061005	AABR07016572.1	0.027773738	-1.108752954
ENSRNOG00000026873	AC136588.1	0.027809533	1.940310911
ENSRNOG00000010514	Cachd1	0.027825967	1.341457393
ENSRNOG00000004676	Pak3	0.028015686	-1.252738599
ENSRNOG00000049900	Irf2bp2	0.028045309	1.44338299
ENSRNOG00000059408	Prr12	0.028130534	1.474620406
ENSRNOG00000015845	Niban2	0.028198883	0.914950412
ENSRNOG00000049552	AABR07030903.1	0.028253215	1.838171406
ENSRNOG00000018778	Cadm1	0.028285041	-0.981693752
ENSRNOG00000003596	Itgb1bp2	0.028292275	-1.093387913
ENSRNOG00000004172	Pdk2	0.028390386	1.643047057
ENSRNOG00000062011	AABR07058511.2	0.028435133	-2.149313021
ENSRNOG00000002312	Atp10d	0.028461834	1.933192692
ENSRNOG00000000161	Chm	0.028524698	1.336796488

ENSRNOG00000059793	Rab37	0.028550186	1.245576305
ENSRNOG00000036697	Mafg	0.02859012	-0.905325906
ENSRNOG00000061515	Fbxo45	0.028636788	1.764784965
ENSRNOG00000057458	Oip5	0.028675559	1.324512896
ENSRNOG00000028814	Oasl2	0.028822402	-0.962986447
ENSRNOG00000013729	RGD1306271	0.02885674	1.276334199
ENSRNOG00000022736	Cdkn2aip	0.028878378	0.793128975
ENSRNOG00000034168	Gemin7l1	0.029044513	2.011959176
ENSRNOG00000011987	Cd2ap	0.029051089	1.051726972
ENSRNOG00000015670	#N/A	0.029089275	1.606935878
ENSRNOG00000006979	Hpca	0.029127415	2.2115712
ENSRNOG00000016033	Endog	0.029221527	1.298167813
ENSRNOG00000038597	Dlg1	0.029437816	1.049132865
ENSRNOG00000060479	Sp3	0.029456324	1.438984065
ENSRNOG00000021338	Tmem132a	0.029490874	1.762680182
ENSRNOG00000009789	Topbp1	0.029535387	1.43798219
ENSRNOG00000000407	Dcbld1	0.029536538	1.266485218
ENSRNOG00000012736	Znrd2	0.029581334	1.339647422
ENSRNOG00000020517	Prrg2	0.029592338	0.804293276
ENSRNOG00000057221	Scn3b	0.029633301	-0.852975232
ENSRNOG00000018109	Clic4	0.029642651	1.596528944
ENSRNOG00000000437	Agpat1	0.029671444	0.801204973
ENSRNOG00000001686	Hlcs	0.029679787	2.141056097
ENSRNOG00000009406	Tm9sf4	0.029899803	0.89204289
ENSRNOG00000043215	Rtbdn	0.029970115	-1.018055117
ENSRNOG00000011544	Zfp219	0.030006062	1.918291526
ENSRNOG00000005905	Pde4b	0.030066448	1.967814354
ENSRNOG00000053502	Arhgef17	0.030090766	3.066441431
ENSRNOG00000055365	Edem1	0.03009661	1.220897644
ENSRNOG00000013431	Rsbn1l	0.030110647	-1.016472363
ENSRNOG00000047867	Klhdc8b	0.030111326	0.897610604
ENSRNOG00000018785	Slc16a13	0.030230744	0.906416002
ENSRNOG00000019907	Nfkbie	0.030289158	1.38678154
ENSRNOG00000037911	LOC680227	0.030311168	1.655599778
ENSRNOG00000032828	Mapk8ip2	0.030332939	0.870360083
ENSRNOG00000028580	Pcnx2	0.03042286	1.823705966
ENSRNOG00000002050	Igfbp7	0.030511797	0.800918012
ENSRNOG00000000042	Xpr1	0.030535385	1.598746466
ENSRNOG00000031129	Carm1	0.030548982	1.698086623
ENSRNOG00000023484	Rpap2	0.030642086	-0.743136084
ENSRNOG00000002423	AABR07029651.1	0.030718993	1.925599711
ENSRNOG00000050210	RT1-CE10	0.030751218	1.09876837
ENSRNOG00000033791	Apc2	0.030774849	0.903989134
ENSRNOG00000001173	Cabp1	0.030874988	-0.835863484

ENSRNOG00000052945	AABR07002969.1	0.030876651	2.005567801
ENSRNOG00000028543	AABR07052523.1	0.030914385	0.786084834
ENSRNOG00000015202	Dnajc5	0.030936396	1.100578419
ENSRNOG00000019336	Gata3	0.03101527	-0.938712375
ENSRNOG00000018557	Cdh22	0.031148105	1.945549766
ENSRNOG00000003834	Map2k4	0.031156204	1.013890708
ENSRNOG00000009930	Pigo	0.031246596	1.317814352
ENSRNOG00000046568	AABR07051791.1	0.031297733	-1.770723707
ENSRNOG00000016849	Ppp2r5d	0.031321972	1.383464437
ENSRNOG00000011887	RGD1307929	0.031350651	0.885236227
ENSRNOG00000011165	Dydc1	0.031389098	-1.220768163
ENSRNOG00000012016	Npc1	0.031427673	1.375187489
ENSRNOG00000059715	Cc2d2a	0.031498984	-0.832230214
ENSRNOG00000028016	AABR07021402.1	0.031523727	-2.283290802
ENSRNOG00000017800	Foxc1	0.031531071	-0.884640049
ENSRNOG00000053790	AABR07036007.1	0.031566858	-1.362789011
ENSRNOG00000032882	#N/A	0.031568688	-1.393702234
ENSRNOG00000050051	Pin4	0.031641021	-1.195973009
ENSRNOG00000020603	Angptl6	0.031685362	-0.758823787
ENSRNOG00000023356	Eif5b	0.031728777	-1.205756879
ENSRNOG00000015534	Cnep1r1	0.031734909	0.91818867
ENSRNOG00000051627	AABR07053580.1	0.031829651	1.168025332
ENSRNOG00000037124	#N/A	0.031856296	-1.644653739
ENSRNOG00000021916	Slc16a12	0.031939514	2.065281692
ENSRNOG00000013089	Kif13b	0.031950271	1.598387685
ENSRNOG00000001792	Slc12a8	0.032113345	0.880302245
ENSRNOG00000005865	Itprid2	0.03227212	1.008561072
ENSRNOG00000061262	Huwe1	0.032456955	1.34087851
ENSRNOG00000047598	Ctdsp2	0.032479692	1.51021619
ENSRNOG00000015903	Add2	0.032498648	1.536305537
ENSRNOG00000010029	Ubald2	0.032505023	1.183275484
ENSRNOG00000039994	Upf3b	0.032543351	-1.342762776
ENSRNOG00000037793	Cdk5r2	0.032551255	1.01409697
ENSRNOG00000008728	Unc79	0.032575191	1.18713825
ENSRNOG00000007427	Entpd6	0.032586129	0.849411036
ENSRNOG00000001864	Tmem191c	0.032656395	1.768732995
ENSRNOG00000061733	LOC103690156	0.032732788	1.120937496
ENSRNOG00000049689	Smim18	0.032799109	-1.434831561
ENSRNOG00000013209	Barhl1	0.032804798	0.916883072
ENSRNOG00000048878	Smim24	0.032820222	1.192925063
ENSRNOG00000052894	Epg5	0.032841616	1.650633675
ENSRNOG00000012095	Pkia	0.032851613	1.053628716
ENSRNOG00000057945	Fancg	0.032865533	1.319779978
ENSRNOG00000011618	Mmaa	0.032940529	-0.762742859

ENSRNOG00000004806	Strn	0.032959043	1.439933376
ENSRNOG00000004726	#N/A	0.033010478	0.997419817
ENSRNOG00000021441	Reln	0.033026999	1.428350329
ENSRNOG00000007359	#N/A	0.033055564	1.25756479
ENSRNOG00000002129	Lrrc8b	0.033079644	1.187203982
ENSRNOG00000016108	Phlpp2	0.033152533	1.632153609
ENSRNOG00000003807	Wnt9b	0.033167968	-1.075140865
ENSRNOG00000006368	Lrrn3	0.033196143	1.234653741
ENSRNOG00000005561	Brinp1	0.033245932	1.105795447
ENSRNOG00000006025	Lamb3	0.0332543	1.323756055
ENSRNOG00000026055	Neurod6	0.033298638	-2.177675823
ENSRNOG00000060726	AABR07072423.1	0.033555198	-2.583891059
ENSRNOG00000024450	Poc1b	0.033575176	-0.823657079
ENSRNOG00000024663	Fkbp15	0.033590744	1.348010412
ENSRNOG00000000417	Numa1	0.033606927	1.054878398
ENSRNOG00000027540	Fam102b	0.033623699	2.323780657
ENSRNOG00000021330	Sprtn	0.033756811	1.729146711
ENSRNOG00000019099	AABR07054578.1	0.033903081	1.539317597
ENSRNOG00000029556	LOC304725	0.034024878	-1.798673035
ENSRNOG00000053240	Soga1	0.034026219	1.972470936
ENSRNOG00000051528	Ddx11	0.034045149	1.760233036
ENSRNOG00000004873	Prkch	0.034101418	2.129089081
ENSRNOG00000032924	AABR07064753.1	0.034151439	-1.834732603
ENSRNOG00000009894	Vps50	0.0341761	1.493851798
ENSRNOG00000004962	Prdm4	0.0341943	1.374254075
ENSRNOG00000014668	RGD621098	0.034235186	1.398185948
ENSRNOG00000003657	Pkmyt1	0.034249197	1.988994715
ENSRNOG00000009033	Cntn2	0.034254932	2.022904904
ENSRNOG00000003924	Pi4k2b	0.034368134	1.615650081
ENSRNOG00000015152	Pkp3	0.034435984	0.947244935
ENSRNOG00000025624	#N/A	0.03449985	-0.862441733
ENSRNOG00000011614	Tmcc1	0.034548614	1.1807734
ENSRNOG00000049952	Gucd1	0.034564932	1.577856597
ENSRNOG00000017642	Acbd5	0.034679483	1.218294608
ENSRNOG00000060931	Rspry1	0.034764745	1.286046253
ENSRNOG00000021265	Cds2	0.034768948	1.111513113
ENSRNOG00000010466	Chpf2	0.034781873	1.396426773
ENSRNOG00000004311	Gpr182	0.034861013	1.853089827
ENSRNOG00000052167	Marveld2	0.034967299	1.098573691
ENSRNOG00000051384	AABR07002337.1	0.035022236	-1.776365545
ENSRNOG00000024454	Ccdc149	0.035040965	1.616856963
ENSRNOG00000051294	AABR07034923.1	0.035062829	-1.774721944
ENSRNOG00000010545	Mrap2	0.035151498	-0.963118505
ENSRNOG00000019306	Syt12	0.035165684	-0.861273328

ENSRNOG00000001548	Nfe2l2	0.035190097	0.768067762
ENSRNOG00000020813	Ltbp3	0.035211614	1.578644593
ENSRNOG00000022595	LOC100362965	0.03521489	1.554303514
ENSRNOG00000024801	#N/A	0.035332733	1.249887227
ENSRNOG00000003901	Cfap36	0.035393153	-0.850053932
ENSRNOG00000016186	Zfp709	0.035453848	0.856570753
ENSRNOG00000000891	#REF!	0.035561871	-1.298223421
ENSRNOG00000034266	Ccdc63	0.035570822	-1.104801186
ENSRNOG00000004169	Fzr1	0.035580875	1.3010359
ENSRNOG00000021183	Rcor2l1	0.035594428	-1.602123747
ENSRNOG00000005811	#N/A	0.035622377	1.330857135
ENSRNOG00000029813	AABR07035218.1	0.035633907	1.200641807
ENSRNOG00000001813	Dnm1l	0.035736397	0.921854978
ENSRNOG00000026271	Tmem245	0.035805969	1.252659283
ENSRNOG00000021026	Zfp687	0.035903605	1.333862219
ENSRNOG00000047781	Slc25a23	0.035943267	2.261575915
ENSRNOG00000046871	LOC681367	0.036182646	1.455704411
ENSRNOG00000016172	Ric1	0.036196331	1.429858182
ENSRNOG00000008934	Tmem65	0.03620232	0.965242928
ENSRNOG00000014838	#N/A	0.036242607	1.242235733
ENSRNOG00000024763	LOC685431	0.036255931	1.223954297
ENSRNOG00000016419	Pdlim5	0.036257762	1.604778024
ENSRNOG00000009005	Slco2a1	0.036330643	0.789952668
ENSRNOG00000018232	Srf	0.036368008	0.822748647
ENSRNOG00000002541	Pds5a	0.036448782	0.815674358
ENSRNOG00000012733	Ankrd12	0.036462441	-0.909564688
ENSRNOG00000054840	AABR07053837.1	0.036598081	-0.94507744
ENSRNOG00000018373	Tln2	0.03664441	1.97107752
ENSRNOG00000008224	Jdp2	0.03674858	1.177514619
ENSRNOG00000046644	Prelid3b	0.036765892	0.85781802
ENSRNOG00000022911	Hjurp	0.036792388	1.371301944
ENSRNOG00000014460	Hivep1	0.036796735	0.860483203
ENSRNOG00000024019	Ccdc6	0.036804427	1.624326521
ENSRNOG00000019573	Lcat	0.036848808	-1.192971486
ENSRNOG00000008245	AABR07054614.1	0.036850292	-1.143873334
ENSRNOG00000060020	C1ql4	0.036862891	1.51857891
ENSRNOG00000014678	Fzd5	0.036888812	1.855136841
ENSRNOG00000012440	Msra	0.036902952	2.216678383
ENSRNOG00000009274	Fut11	0.03693351	1.457289767
ENSRNOG00000033057	AABR07025010.1	0.036999163	1.023979687
ENSRNOG00000021102	#N/A	0.03702617	1.44928671
ENSRNOG00000003785	Usp43	0.037056654	1.902138273
ENSRNOG00000007030	Epha7	0.037192247	0.901861348
ENSRNOG00000060614	Pxdn	0.037280405	1.615327986

ENSRNOG00000002940	Ankrd17	0.037342718	-0.905756109
ENSRNOG00000021021	Ffar2	0.03737122	1.472399829
ENSRNOG00000021041	#N/A	0.037412883	1.2972858
ENSRNOG00000004760	Lars2	0.037422111	1.678086049
ENSRNOG00000008782	Pnisr	0.03743917	-0.971313819
ENSRNOG00000045647	Hax1	0.037616853	1.41273947
ENSRNOG00000054603	Pacsin1	0.037637022	1.989186243
ENSRNOG00000053560	Rhou	0.037652978	1.88243686
ENSRNOG00000013178	#N/A	0.037699194	1.420021401
ENSRNOG00000005070	Spopl	0.037707539	1.524794628
ENSRNOG00000018582	Exosc6	0.037731169	1.44024374
ENSRNOG00000057806	Trpm7	0.037862827	1.080883454
ENSRNOG00000015333	AABR07070225.1	0.037877196	-0.943691749
ENSRNOG00000056526	AABR07054262.1	0.03795153	1.426487896
ENSRNOG00000036683	Sirt7	0.037962811	0.801739806
ENSRNOG00000018110	Svil	0.037997009	0.795907714
ENSRNOG00000037798	Erich4	0.038019924	-1.515696009
ENSRNOG00000017899	Akr7a3	0.038038805	-1.093960682
ENSRNOG00000014434	Chd1	0.038039156	0.812761574
ENSRNOG00000050057	Abca3	0.038064419	1.409621912
ENSRNOG00000004749	Slc30a1	0.0380728	0.906758574
ENSRNOG00000061254	AABR07015042.1	0.038074446	-2.866982533
ENSRNOG00000008869	Ppp1r9a	0.038121874	1.057666146
ENSRNOG00000009057	Sec62	0.038138766	-1.244062306
ENSRNOG00000025174	#N/A	0.038185239	1.066858628
ENSRNOG00000012163	#N/A	0.038209075	1.745462027
ENSRNOG00000033411	Usp12	0.038334149	0.891010015
ENSRNOG00000020715	Ddb1	0.03833431	0.880361164
ENSRNOG00000059440	AABR07068316.2	0.03837918	-1.775715283
ENSRNOG00000005923	Mtmr2	0.038587074	0.973463034
ENSRNOG00000019552	Lsm7	0.038632561	-0.788267505
ENSRNOG00000028436	Rprml	0.038666561	-0.810386477
ENSRNOG00000015430	Nlgn2	0.038842417	1.408450187
ENSRNOG00000010750	Twistnb	0.038896797	-1.054559366
ENSRNOG00000002255	Fam162a	0.038925006	-0.750661368
ENSRNOG00000006515	Klhl15	0.038941279	1.160085278
ENSRNOG00000061519	Asap2	0.039029827	1.597195415
ENSRNOG00000024566	Sh3d21	0.039048099	-0.993726261
ENSRNOG00000009836	Rbm26	0.039141861	-1.201903359
ENSRNOG00000020272	5330417C22Rik	0.039201702	0.815647153
ENSRNOG00000001781	Lmln	0.039332827	1.092974458
ENSRNOG00000007975	Ncoa2	0.039394783	1.692676602
ENSRNOG00000050510	Rab1b	0.0393948	0.766447431
ENSRNOG00000011329	Pkm	0.039418505	0.985778303

ENSRNOG00000046424	Mfn2	0.039454908	1.157810888
ENSRNOG00000033235	Relb	0.039617932	1.33588257
ENSRNOG00000027022	AABR07043654.1	0.039645794	1.614228302
ENSRNOG00000039476	Pcdhb2	0.039661361	1.758908684
ENSRNOG00000018003	F2rl1	0.039672746	0.902924396
ENSRNOG00000013169	Traf4	0.039754098	0.932184967
ENSRNOG00000010921	Taf1d	0.039769344	-1.158059683
ENSRNOG00000023917	#N/A	0.039800863	1.461229368
ENSRNOG00000048213	AABR07039304.1	0.03986113	1.207101816
ENSRNOG00000030404	Arrb1	0.039898659	1.682209527
ENSRNOG00000019857	Gng7	0.039904487	1.315578436
ENSRNOG00000018881	Rnasek	0.040053629	0.825550883
ENSRNOG00000000036	Klhdc8a	0.040155511	0.768461475
ENSRNOG00000059043	Itch	0.040158112	1.31934802
ENSRNOG00000005087	Cebpz	0.040180949	-0.895723354
ENSRNOG00000056332	Miga1	0.040191335	1.420562984
ENSRNOG00000056297	Fmnl3	0.040196988	1.715589328
ENSRNOG00000023896	Dusp6	0.04022238	-1.148245851
ENSRNOG00000008683	Alk	0.040385823	-0.920065048
ENSRNOG00000006796	LOC102555183	0.040466609	1.385799713
ENSRNOG00000017951	Ranbp9	0.040482099	0.798182248
ENSRNOG00000009106	C2cd4b	0.040496202	1.388139929
ENSRNOG00000002229	Adcy5	0.040518821	1.148645243
ENSRNOG00000016984	Atg13	0.04053056	2.323046604
ENSRNOG00000010846	#N/A	0.040690429	1.599754692
ENSRNOG00000010934	Spa17	0.040732057	-1.081516606
ENSRNOG00000032258	Swt1	0.040809583	-0.839687155
ENSRNOG00000056036	LOC103691005	0.040832109	-1.106144404
ENSRNOG00000045698	Lin7c	0.04090965	0.945606876
ENSRNOG00000050424	Nme4	0.040934514	0.937763558
ENSRNOG00000017857	LOC108348250	0.041154328	1.433748526
ENSRNOG00000003251	B3galt2	0.041366725	1.347924558
ENSRNOG00000046891	Hk1	0.041431393	1.430538362
ENSRNOG00000054775	Fkbp11	0.041458012	0.796434044
ENSRNOG00000009613	Sh3bgrl2	0.041680901	1.425413691
ENSRNOG00000008754	Flvcr2	0.041722422	-1.263591854
ENSRNOG00000019579	Edc3	0.041867091	1.285570019
ENSRNOG00000060571	Tmem189	0.041998953	0.876028102
ENSRNOG00000007645	Kcnj9	0.042062098	-0.841650754
ENSRNOG00000020115	Ilkap	0.042143204	-0.73748489
ENSRNOG00000042333	Dnal1	0.042180616	-0.82521789
ENSRNOG00000062175	AABR07034274.1	0.042191764	-2.003260531
ENSRNOG00000008239	Repin1	0.0422062	1.403441943
ENSRNOG00000011140	Prxl2a	0.042313581	-0.788713222

ENSRNOG00000013170	Il13ra1	0.042477084	1.611506385
ENSRNOG00000010543	Srpra	0.042536316	0.897962253
ENSRNOG00000057558	AC128792.2	0.042538612	1.498839066
ENSRNOG00000014006	Neto1	0.042549054	-1.914186813
ENSRNOG00000050453	AABR07002848.1	0.042667888	2.681084639
ENSRNOG00000003707	Zmym3	0.042694245	1.02890277
ENSRNOG00000007632	Mss51	0.042785844	-0.973701443
ENSRNOG00000015753	Epn1	0.042992191	1.159657557
ENSRNOG00000020843	#N/A	0.043059121	1.023457201
ENSRNOG00000028592	AABR07035074.1	0.043101757	1.092793815
ENSRNOG00000050091	Efcab1	0.043131116	2.389854495
ENSRNOG00000031135	Smarcc2	0.043203013	1.06998698
ENSRNOG00000015434	Midn	0.043288791	0.973873055
ENSRNOG00000038905	AABR07048397.1	0.043638086	1.489373543
ENSRNOG00000059055	Hsbp1l1	0.043661294	-1.230749193
ENSRNOG00000020525	Col5a3	0.043818021	-0.948961718
ENSRNOG00000019075	Stat5b	0.043818321	1.368814402
ENSRNOG00000009936	Dido1	0.043858813	0.917012871
ENSRNOG00000061785	AABR07065124.2	0.043867546	-1.736735098
ENSRNOG00000011794	Tcea3	0.043975706	-1.317487112
ENSRNOG00000057601	AABR07044080.2	0.043998856	-1.104191
ENSRNOG00000033169	Cpeb4	0.044089938	1.539642705
ENSRNOG00000052528	SNORA73	0.044111458	-1.66097168
ENSRNOG00000001931	Fgf12	0.044129813	-0.891142845
ENSRNOG00000023721	LOC502684	0.044134339	-1.091457089
ENSRNOG00000016316	Mcm2	0.044142337	1.009695657
ENSRNOG00000010156	Snapc5	0.044325419	0.958470508
ENSRNOG00000031778	Mef2d	0.044330635	0.846455861
ENSRNOG00000008247	Ikbip	0.044414146	-0.882980749
ENSRNOG00000015026	Arhgef11	0.044469144	1.308067094
ENSRNOG00000056617	Zswim8	0.044534633	2.030220652
ENSRNOG00000055750	AABR07044717.1	0.044607093	2.015677057
ENSRNOG00000018416	Ttbk1	0.044785036	1.21678919
ENSRNOG00000027259	AABR07029661.1	0.044789836	1.356299274
ENSRNOG00000009977	Thrap3	0.044940702	0.968404423
ENSRNOG00000007300	C1qtnf6	0.04497365	0.968644354
ENSRNOG00000039740	Cenpk	0.044991464	-1.235862399
ENSRNOG00000017858	Tsr3	0.045032237	1.295270141
ENSRNOG00000010875	Fam241a	0.045035983	0.7758888
ENSRNOG00000059579	Gpt2	0.045092003	1.529744255
ENSRNOG00000027436	Zfp324	0.045119287	1.754635567
ENSRNOG00000011661	Hnrnph2	0.045119625	1.063724984
ENSRNOG00000008332	Smo	0.04516521	-1.613867168
ENSRNOG00000027229	Slc35d2	0.045176079	-0.860600237

ENSRNOG00000014513	Rexo5	0.045217876	-0.746463269
ENSRNOG00000025523	Armh3	0.045233804	1.249981707
ENSRNOG00000010486	Mpp4	0.045325793	-1.269988716
ENSRNOG00000005447	Ypel2	0.045473503	1.572006751
ENSRNOG00000020134	Upf1	0.045549483	1.310428748
ENSRNOG00000042536	Pde4d	0.045604107	1.109981848
ENSRNOG00000001130	Nos1	0.045630644	2.068974486
ENSRNOG00000005391	Prex2	0.045715373	-0.870364144
ENSRNOG00000054429	AABR07011697.1	0.045744322	1.577541897
ENSRNOG00000020773	Map3k11	0.045756211	1.022836907
ENSRNOG00000017837	Znf48	0.045899514	1.364267266
ENSRNOG00000008036	Dennd4c	0.045910863	1.3315165
ENSRNOG00000028025	Aknad1	0.045969984	1.679029667
ENSRNOG00000047225	Tma7	0.046038705	-0.782112582
ENSRNOG00000024602	Plekha7	0.046130137	2.044631067
ENSRNOG00000000693	#N/A	0.046150974	1.465849806
ENSRNOG00000061524	AABR07068285.2	0.046163986	1.615215703
ENSRNOG00000015002	Abhd15	0.046203	1.243111977
ENSRNOG00000015911	Lrp5	0.046209663	0.791525926
ENSRNOG00000016391	Arid4b	0.046242381	-1.432262426
ENSRNOG00000043060	Dnajc4	0.04629055	-0.821332139
ENSRNOG00000018113	AABR07028352.1	0.046306759	0.996454428
ENSRNOG00000033346	LOC100361636	0.046323684	-1.814892469
ENSRNOG00000016606	Snorc	0.046448479	-1.647196834
ENSRNOG00000062091	AC116220.2	0.046496003	1.78336609
ENSRNOG00000020423	Apc	0.046512638	0.945880427
ENSRNOG00000012806	Rbbp6	0.046548334	-0.896566796
ENSRNOG00000025332	Cd109	0.046569025	-1.325744345
ENSRNOG00000013663	Tmem86a	0.046579486	0.848619615
ENSRNOG00000003463	Srebf1	0.046623212	1.27172559
ENSRNOG00000008425	Nav1	0.046690253	1.399975253
ENSRNOG00000023536	Adgrd1	0.046707575	1.554051688
ENSRNOG00000005183	Rtf1	0.046767374	-0.863133278
ENSRNOG00000032327	Pdia5	0.046778474	-0.74095816
ENSRNOG00000007097	Gpatch3	0.046918958	1.70006843
ENSRNOG00000027728	Ibtk	0.046991986	0.829764116
ENSRNOG00000017523	H6pd	0.04699258	1.1326455
ENSRNOG00000004563	Sec24a	0.047140775	1.337728233
ENSRNOG00000002585	Cul4b	0.047305856	-1.083624954
ENSRNOG00000008113	RGD1561149	0.047408228	1.122768373
ENSRNOG00000009683	Sdcbp	0.047569866	-0.922629988
ENSRNOG00000009965	Pih1d2	0.047614072	-1.005445292
ENSRNOG00000010895	Tmem30a	0.047662026	0.873908447
ENSRNOG00000050978	LOC100911295	0.047684957	0.846003672

ENSRNOG00000023086	LOC100360828	0.04771596	-1.046545453
ENSRNOG00000052087	Ubap2	0.047853255	1.037222429
ENSRNOG00000046405	Hic1	0.047911187	-1.068819635
ENSRNOG00000012420	Bcl9l	0.04803852	1.518278241
ENSRNOG00000000961	#N/A	0.048056022	0.874052817
ENSRNOG00000020289	Akt1s1	0.048072115	0.787029008
ENSRNOG00000026143	Ckap2l	0.048240775	1.176629648
ENSRNOG00000009653	Numb	0.048252856	1.142388576
ENSRNOG00000053654	AABR07061614.3	0.048293392	1.369661209
ENSRNOG00000046803	Arhgef16	0.048325281	0.821432215
ENSRNOG00000026519	LOC100364062	0.048367925	1.238577759
ENSRNOG00000018475	#N/A	0.04845094	1.03324472
ENSRNOG00000018980	Tjap1	0.048558734	0.876708685
ENSRNOG00000060946	Cask	0.048570376	1.355096708
ENSRNOG00000033496	Igdcc4	0.048585247	2.136812122
ENSRNOG00000047495	AABR07049948.1	0.048721696	1.386011558
ENSRNOG00000013314	Avl9	0.0487437	1.231686088
ENSRNOG00000058515	AABR07054460.4	0.048807952	2.37270361
ENSRNOG00000060594	LOC100911672	0.048842643	1.065139613
ENSRNOG00000003694	Prox1	0.048872789	0.95291863
ENSRNOG00000050343	Jmy	0.048916608	1.795056301
ENSRNOG00000018066	Bccip	0.048919147	0.795173221
ENSRNOG00000020426	#N/A	0.049367678	0.951243349
ENSRNOG00000036677	Slc16a3	0.049434122	1.19289643
ENSRNOG00000056212	Flt3lg	0.049494629	-0.937439372
ENSRNOG00000038004	Zfp804a	0.049580607	1.03322235
ENSRNOG00000016294	Cd4	0.049643077	1.735970624
ENSRNOG00000056770	AABR07002875.1	0.049652757	-1.399211904
ENSRNOG00000009110	Psen1	0.049664213	1.031053316
ENSRNOG00000027949	Rfx6	0.049705654	-0.848689585
ENSRNOG00000015953	Oaz2	0.049711603	1.135045831
ENSRNOG00000051564	Rap2a	0.049744421	1.076566104
ENSRNOG00000008658	Mitf	0.04983322	-1.219448184
ENSRNOG00000050860	Abcb11	0.04984946	-1.212798916
ENSRNOG00000002021	Son	0.049908408	-0.739601377
ENSRNOG00000033202	Fbxo41	0.049929639	1.927258332
ENSRNOG00000018714	Arl5b	0.049943607	-0.856134166
ENSRNOG00000021105	Gabpb2	0.04995346	1.031974398
ENSRNOG00000019645	Osbp2	0.049984021	0.886463663

Supplementary Table S4. NCBI gene IDs used in investigation of gene expression by qPCR.

Gene name	NCBI I.D
Ins1	NM_019129.3
Ins2	NM_019130.2
Tgs1	NM_001107904.1
MafA	NM_001399773.1
GAPDH	NM_017008.4
Pdx1	NM_022852.4
Hdac5	NM_053450
Munc18	NM_013038
NeuroD1	NM_019218
PRIP	NM_001276714
SNAP25	NM_001270576
Pax6	NM_013001.2
Gnas	NM_001024823.4
Kcnj11	NM_031358.3
Gpr119	NM_181770.1
Kcnn1	NM_019313.1
Rab3A	NM_013018.2
Gpi	NM_207592.1
Klf11	NM_001037354.2
Nr4A1	NM_024388.2
Syt11	NM_031667.3
Syt13	NM_030839.3
